# Anthropogenic perturbations to atmospheric methane reflected in Greenland firn air clumped isotope measurements

**DOI:** 10.1126/sciadv.aeb2203

**Published:** 2026-07-15

**Authors:** Malavika Sivan, Jiayang Sun, Patricia Martinerie, Maria Elena Popa, James Farquhar, Maarten Krol, Carina van der Veen, Bibhasvata Dasgupta, Mojhgan A. Haghnegahdar, Camilla Marie Jensen, Ji-Woong Yang, Johannes Freitag, Kévin Fourteau, Thomas Blunier, Thomas Röckmann

**Affiliations:** ^1^ Institute for Marine and Atmospheric Research, Utrecht University, 3584 CC Utrecht, Netherlands.; ^2^ Department of Geology, University of Maryland, College Park, MD 20742, USA.; ^3^ Air Resources Lab, National Oceanic and Atmospheric Administration, College Park, MD 20740, USA.; ^4^ IGE, Université Grenoble Alpes, CNRS, INRAE, IRD, Grenoble INP, 38000 Grenoble, France.; ^5^ Earth System Science Interdisciplinary Center, University of Maryland, College Park, MD 20740, USA.; ^6^ Physics of Ice Climate and Earth, Niels Bohr Institute, University of Copenhagen, Tagensvej 16, 2200 Copenhagen, Denmark.; ^7^ Laboratoire des Science du Climat et de l'Environnement, 91191 Gif-sur-Yvette, France.; ^8^ Alfred-Wegener-Institute, 27570 Bremerhaven, Germany.

## Abstract

The ongoing debate over the origin of rising atmospheric methane levels highlights gaps in our understanding of its global budget. To our knowledge, we present the first reconstructed clumped isotopic composition of atmospheric methane dating back to the early 1990s, based on large-volume firn air samples from the Greenland ice cap. Our measurements indicate that atmospheric ∆^12^CH_2_D_2_ around 1993 was 10 ± 2 per mil lower than in the mid-2020s, likely marking the lowest level over the last millennium. We attribute this shift to the unprecedented anthropogenic perturbation of global methane emissions during the industrial period. The temporal lag in the atmospheric isotopic signature reflects the disequilibrium effect caused by the longer atmospheric lifetime of ^12^CH_2_D_2_ relative to methane (CH_4_). Moreover, our results suggest that the kinetic isotope effect in the removal of ^12^CH_2_D_2_ by the hydroxyl radical needs to be much smaller than previously estimated. Future changes in atmospheric ∆^12^CH_2_D_2_ will continue to reflect evolving CH_4_ emission and removal pathways.

## Teaser

Greenland firn reveals unprecedented human-driven shift in atmospheric methane isotopes since the 1990s.

## INTRODUCTION

Rising methane (CH_4_) emissions since preindustrial times have contributed ~0.5°C to global warming and continue to intensify climate change (*1*). The long-term rise of atmospheric CH_4_ was interrupted by a brief stagnation in the early 2000s, followed by an accelerating growth since 2007 (*2*, *3*). Understanding the underlying drivers of these changes is crucial for efforts to slow down and eventually reverse the increase. Measurements of the bulk isotopic composition of CH_4_ (δ^13^C and δD) are valuable tools for identifying which processes (e.g., emissions from fossil fuels, agriculture, waste, natural wetlands, or changes in atmospheric removals) drive atmospheric CH_4_ upward and how these processes change over time. However, given the multitude of anthropogenic and natural drivers, the problem is underconstrained, and consensus regarding the factors controlling atmospheric CH_4_ concentrations has yet to be reached (*4*–*8*).

Additional information about CH_4_ sources and sinks can be derived from CH_4_ molecules with two heavy isotopes, ^13^CH_3_D and ^12^CH_2_D_2_, the so-called clumped isotopes of CH_4_. The clumped isotopic composition, denoted as Δ^13^CH_3_D and Δ^12^CH_2_D_2_, quantifies the difference between the measured and statistically expected abundance of these doubly substituted isotopologues (equations 3 and 4 in the Supplementary Materials). Measurements indicate that microbial CH_4_ generally exhibits much lower Δ^12^CH_2_D_2_ than fossil CH_4_ (*9*, *10*). Conversely, the Δ^12^CH_2_D_2_ values of ambient air CH_4_ are significantly higher than those of any known major CH_4_ source, implying a strong influence from atmospheric sinks (*11*–*14*). However, Sivan *et al.* (*13*) hypothesized that the theoretical values of the kinetic isotope effect (KIE) in the removal of the clumped isotopologues, especially ^12^CH_2_D_2,_ are overestimated, highlighting the need for additional atmospheric CH_4_ measurements to better constrain these KIEs.

To date, our understanding of the clumped isotopologue signature of atmospheric CH_4_ is restricted to (*11*, *13*, *14*), and how the clumped isotopic composition of atmospheric CH_4_ has evolved over time remains unconstrained. This knowledge gap primarily stems from the large air sample volumes required to measure CH_4_ clumped isotopologue signals, given the rarity of these doubly substituted isotopologues. No available air archives can provide the large sample volumes required. Firn-trapped air overcomes this limitation: Firn, the transitional stage between snow and glacial ice, contains an interconnected pore network that allows air to mix within layers, preserving a time-averaged record of past atmospheric composition in sufficient quantities for analysis. In this study, we present the CH_4_ clumped isotopic analysis of firn-trapped air samples collected from the East Greenland Ice-Core Project (EastGRIP) site. Detailed descriptions of the sampling methodology and laboratory analytical procedures are provided in text S2.

## RESULTS AND DISCUSSION

### Reconstruction of atmospheric CH_4_ clumped isotopic composition in the past

**Fig. 1.**
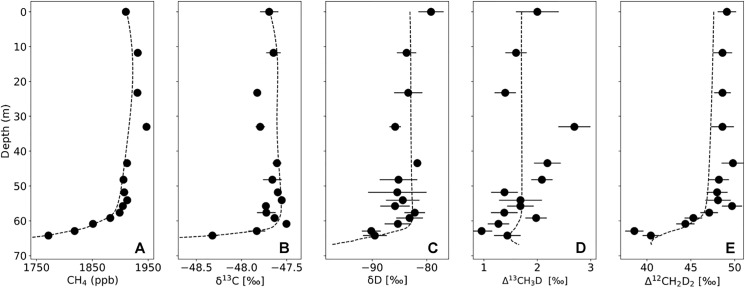
CH_4_ mole fractions and isotopic compositions from firn air samples collected at various depths. (**A**) CH_4_ mole fraction, (**B**) δ^13^C, (**C**) δD, (**D**) Δ^13^CH_3_D, and (**E**) Δ^12^CH_2_D_2_. The black dashed lines show results from the Institut des Géosciences de l'Environnement-Grenoble Images Parole Signal Automatique laboratoire (IGE-GIPSA) firn model (details in text S3) driven by the high-latitude Northern Hemisphere (NH) trend for CH_4_ mole fraction and the atmospheric model output presented in Fig. 2.

Figure 1 shows depth profiles of CH_4_ mole fraction, bulk, and clumped isotope signatures measured on large-volume (300 to 700 liters) firn air samples collected at the EastGRIP site in 2018, along with firn model simulations (details in texts S2 and S3 and tables S1 and S2). The CH_4_ mole fraction increases by about 150 parts per billion (ppb) from the lowest firn layer depth (~65 m) to the surface (0 m). This is accompanied by an increase of ~0.6‰ in δ^13^C and ~6‰ in δD. The measurement uncertainty for Δ^13^CH_3_D is insufficient to resolve a possible vertical gradient. In contrast, Δ^12^CH_2_D_2_ values show a clear and significant increase of ~10‰ in the firn column from the lowest depths to the surface, far larger than typical measurement uncertainties of 1 to 1.4‰ (1 SD).

**Fig. 2.**
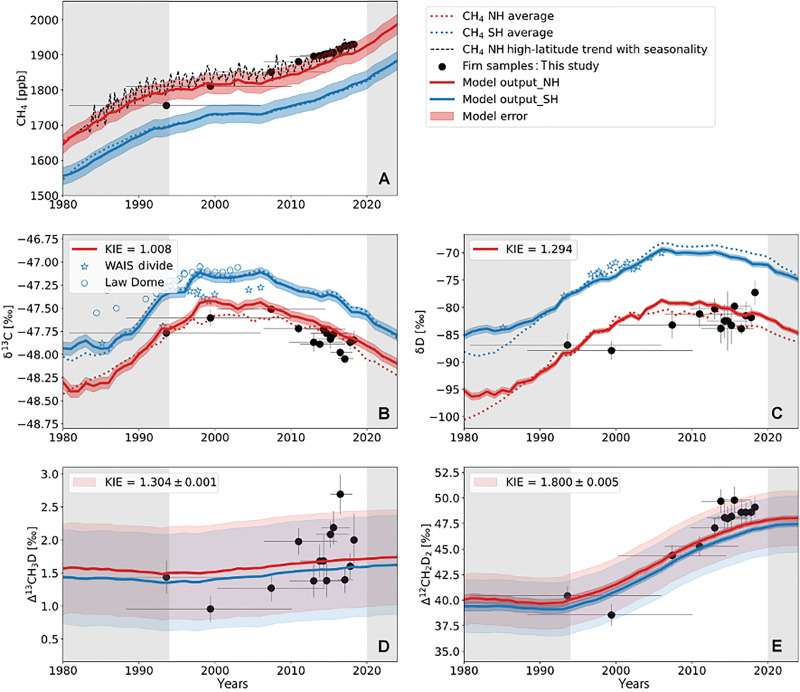
Temporal evolution of atmospheric CH_4_ mole fraction and isotopic signatures for the Southern Hemisphere and NH. (**A**) CH_4_ mole fraction, (**B**) δ^13^C, (**C**) δD, (**D**) Δ^13^CH_3_D, and (**E**) Δ^12^CH_2_D_2_. Black circles: Measurements from this study, vertical gray lines show measurement uncertainties, and horizontal gray error bars represent gas age width (15 to 85% probability range) in firn (details in fig. S1). Blue circles and stars: Measurements from previous studies for the SH and WAIS Divide (*4*, *20*). Red and blue solid lines: Model output from 1980 to 2024 of the two-box forward model using source emission fluxes detailed in text S4 for the NH and SH. The dark shades in (A) to (E) show the uncertainty of the inverse model (*20*), and the light shades in (D) and (E) show the uncertainties in the sink KIE (±0.001 for Δ^13^CH_3_D and ±0.005 for Δ^12^CH_2_D_2_). Red and blue dash-dotted lines in (A) to (C): NH and SH averages calculated from observations at atmospheric monitoring stations, respectively (*20*). Black dashed line in (A): High-latitude CH_4_ trend used for comparison with firn data (see text S3). The gray-shaded areas mark the spin-up and spin-down periods used in the inversion model (*20*). The sink reaction KIEs (same for both NH and SH) for the respective isotopologues are given as legends. SH, Law Dome; WAIS, West Antarctic Ice Sheet.

The measurement results were corrected for diffusion and gravitational settling in the firn layers (details in text S3 and table S2) and converted into a temporal atmospheric history by assigning mean ages (age distributions are shown in fig. S1) (Fig. 2). This was done using a gas transport forward model (*15*) driven by the atmospheric trends presented in Fig. 2. The CH_4_ mole fraction, δ^13^C, and δD retrieved from this work are consistent with previous measurements (*16*, *17*). The large increase (~10 ± 2‰) in Δ^12^CH_2_D_2_ observed from 1993 to 2018 is broadly consistent with a recent model prediction (*11*) but deviates from earlier model results (*18*, *19*).

### The atmospheric CH_4_ isotope budget model for 1980–2024

To investigate this further, we performed simulations with a two-box atmospheric model (details in text S4). The total CH_4_ source emission flux is partitioned into five categories: wetlands, waste, fossil, pyrogenic, and agriculture (see text S5), each with representative isotopic and isotopologue compositions (text S6 and table S3). The emission fluxes for 1980–2024 were derived from an inversion model (*20*) constrained by atmospheric observations of CH_4_, δ^13^C, and δD (text S4 and fig. S5) in this period. The two-box model reproduces both newly reported and previously published Northern Hemisphere (NH) and Southern Hemisphere (SH) observations of CH_4_, δ^13^C, and δD between 1980 and 2024 (Fig. 2). The model also captures the observed ~10 ± 2‰ increase in Δ^12^CH_2_D_2_ of atmospheric CH_4_ from 1990 to 2024.

**Fig. 3.**
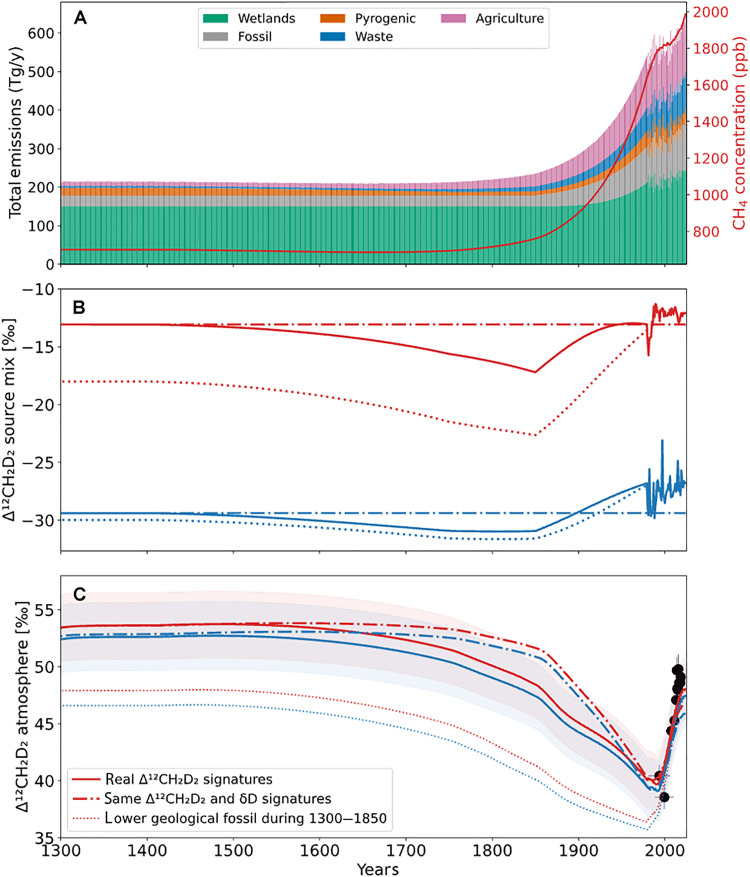
Results of the historical (1300–2025) simulations of CH_4_ emission fluxes, atmospheric CH_4_, hemisphere-specific average source Δ^12^CH_2_D_2_, and atmospheric Δ^12^CH_2_D_2_. (**A**) Total annual emission fluxes from five categories (see text S4) and the atmospheric CH_4_ mole fraction in parts per billion. (**B**) Model-simulated Δ^12^CH_2_D_2_ signature of the global average CH_4_ source. (**C**) Model-simulated Δ^12^CH_2_D_2_ of atmospheric CH_4_ (red: NH; blue: SH) together with our new measurements from Greenland firn air (black circles).

To identify the underlying causes of this substantial change of ~10 ± 2‰ in Δ^12^CH_2_D_2_ of atmospheric CH_4_ from 1990 to 2024, we carried out a wide range of model simulations varying individual and multiple source and sink parameters. The results demonstrate that a Δ^12^CH_2_D_2_ variation of this magnitude cannot be explained by recent changes in CH_4_ sources (figs. S6 and S7). The Δ^12^CH_2_D_2_ of the globally averaged source only changed by less than 1‰ over 1980–2020 (solid lines in Fig. 3B), one order of magnitude less than the observed change. How can the unexpected Δ^12^CH_2_D_2_ trend then be explained? Tans (1997) first showed that the effect of changes in the total CH_4_ sources is much more long lived for the isotopic composition than for the total mole fraction. This is because the global atmospheric CH_4_ mole fraction reaches a new steady state relatively rapidly, as the sinks reach a new equilibrium with the sources. In contrast, the isotope budget takes longer to balance because it requires the entire atmospheric reservoir to be processed by sink reactions under the new conditions. The longer atmospheric lifetime of ^12^CH_2_D_2_ (~20 years) compared to the other CH_4_ isotopologues (~14.3, ~14.2, ~11.1, and 11.0 years, respectively, for ^13^CH_3_D, ^12^CH_3_D, ^13^CH_4_, and ^12^CH_4_) (*18*) leads to an even slower equilibration of the Δ^12^CH_2_D_2_ signal in response to source-sink perturbations. In addition, as the individual stable isotope signatures (δ^13^C and δD) are still evolving, the clumped isotope signature still needs to adjust after new bulk isotope equilibrium has been reached. As a result, there is a temporal lag between changes in CH_4_ concentration (and bulk isotopic composition) and shifts in Δ^12^CH_2_D_2_. Thus, the observed Δ^12^CH_2_D_2_ trend from 1990 to 2018 may be reflecting changes in CH_4_ sources or sinks that occurred further back in the past.

### The atmospheric CH_4_ isotope budget model for 1300–2025

To understand the origin of the ~10 ± 2‰ increase in Δ^12^CH_2_D_2_ signal, it is necessary to analyze the model further back in time. For the period 1000–1730, emission fluxes were taken and slightly modified (less pyrogenic, more geological fossil emissions) from (*21*) to match published ice-core measurements of CH_4_, δ^13^C, and δD. This period remains poorly constrained due to the limited number of available measurements, and implications will be further discussed below. For the period 1730–1980, the fluxes were interpolated with a smooth exponential evolution for all source categories to align with the known CH_4_ mole fraction, δ^13^C, and δD changes (*21*).

The long-term (1000–2024) simulations reproduce the observed CH_4_ trend and satisfy the bulk isotope constraints from (*21*) (fig. S8 and Fig. 3) over the entire millennium. The changes in the total bulk CH_4_ emission fluxes (Fig. 3A) produce a strong long-term decrease in atmospheric Δ^12^CH_2_D_2_ from ~53‰ in 1400 to a minimum of ~40‰ in the 1980s (Fig. 3C). However, the changes in the total source Δ^12^CH_2_D_2_ are much smaller, roughly 2‰ in the SH and 4‰ in the NH (Fig. 3B), which originates from a decrease in pyrogenic emissions (more positive in Δ^12^CH_2_D_2_) and an increase in agricultural emissions (more negative in Δ^12^CH_2_D_2_) between 1450 and 1850, required to reproduce the observed δ^13^C and δD values (*21*). The remaining, larger part of the atmospheric Δ^12^CH_2_D_2_ decrease must originate from deviations in the source-sink equilibrium. When sources outweigh the sinks, the atmospheric isotopic composition shifts toward that of the sources, as the atmospheric reservoir has undergone less fractionation by sinks (*22*). The effect of source-sink imbalance is illustrated by a sensitivity simulation (dash-dotted lines in Fig. 3, B and C) in which the global average source signatures (δD and Δ^12^CH_2_D_2_) are held constant. Despite the fixed source composition, a decrease in atmospheric Δ^12^CH_2_D_2_ is still observed. The difference between the solid and dash-dotted lines in Fig. 3C then represents the effect of changes in the average source signature, as shown in Fig. 3B.

The acceleration of emission rates over the 20th century continuously exacerbated the source-sink disequilibrium, leading to progressively lower Δ^12^CH_2_D_2_ (Fig. 3C). Our model simulations thus demonstrate that the novel tracer Δ^12^CH_2_D_2_ recorded the strong human perturbation of the global CH_4_ cycle over the past centuries. After 1990, the CH_4_ growth rate decreased, allowing the sinks to “catch up” with the sources and restoring Δ^12^CH_2_D_2_ toward higher values (Fig. 3C). The renewed increase since 2007 has not had sufficient time to lead to a renewed decrease in Δ^12^CH_2_D_2_. As shown below, it may take until the end of the century to reestablish isotopic equilibrium under the new global source mix.

We ran our model with different emission fluxes for the different source categories to find a scenario that optimally reproduces the published ice-core measurements and the firn air data in this study. Three different Emissions Database for Global Atmospheric Research (EDGAR) emission scenarios (v6, v7, and v8) were also included to illustrate how the model responds to varying source balances across inventory versions and to evaluate which, if any, are consistent with the observations (fig. S10).

The optimized final emission fluxes for 1300–2024 presented in Fig. 3A include a high fraction of geological (fossil) emissions in the preindustrial atmosphere. This is supported by present-day measurements of geological emissions (*23*, *24*) but is questioned by reconstructions of ^14^CH_4_ (*25*, *26*). A scenario where pyrogenic sources dominate over geological sources between 1000 and 1700, as proposed in (*21*), was also tested (dotted lines in Fig. 3, B and C). The strong Δ^12^CH_2_D_2_ decrease until 1980 is also captured in that scenario, but the preindustrial Δ^12^CH_2_D_2_ values are different. Thus, measurements of Δ^12^CH_2_D_2_ in ice-core air might help resolve the conundrum on preindustrial geological emissions, with important implications for the interpretation of the present-day fossil CH_4_ emissions.

The model simulations, along with our improved understanding of the underlying drivers, suggest that we may have sampled and measured air with the lowest Δ^12^CH_2_D_2_ values (about 40 ± 2‰; Fig. 3) in the recent 1000 years of Earth’s history. This minimum is a result of the unprecedented anthropogenic perturbation of the CH_4_ emissions since the 1850s and the slowdown in the CH_4_ growth rate at the end of the 20th century.

### New constraints on the KIE of atmospheric ^12^CH_2_D_2_ sink

Three key parameters control the clumped isotope budget of atmospheric CH_4_: (i) the isotopic compositions of the individual source categories, (ii) the kinetic fractionation of the sink reactions, and (iii) the source-sink disequilibrium. To constrain the source compositions, we compiled and categorized all the published CH_4_ clumped isotopic composition measurements to estimate the Δ^13^CH_3_D and Δ^12^CH_2_D_2_ of each source category. The resulting flux-weighted global average source signatures for the present atmosphere are around 2.2‰ (SH) and 3.2‰ (NH) for Δ^13^CH_3_D and −29.4‰ (SH) and −13.1‰ (NH) for Δ^12^CH_2_D_2_ (details in text S6 and table S3).

Using these estimates of the source signatures, the new firn air measurements, and the flux imbalances derived from the CH_4_ mole fraction increase (Fig. 3A), the KIEs in the removal reactions can be determined. Fitting the measurements in our simulations requires the total sink fractionations of 304 ± 1‰ for ^13^CH_3_D and 800 ± 21‰ for ^12^CH_2_D_2_ (including source signature uncertainties) (Fig. 2, D and E). For ^13^CH_3_D, this value is close to the product of KIEs of the single substituted isotopologues ^13^CH_4_ (8‰) and ^12^CH_3_D (294‰), as 1.008 × 1.294 = 1.304. This means that the sink has minimal effect on atmospheric Δ^13^CH_3_D values. In contrast, the isotope effect for ^12^CH_2_D_2_ (800‰) is much larger than the product of the KIEs of two single D-substituted isotopologues (1.294 × 1.294 = 1.674). The sink effect on Δ^12^CH_2_D_2_ is then ~1.800/1.673 = ~1.075, meaning that the Δ^12^CH_2_D_2_ of atmospheric CH_4_ is ~75‰ enriched relative to the global average source signature under source-sink equilibrium, in good agreement with observations (*11*–*14*). When sources and sinks are out of equilibrium, atmospheric CH_4_ is more (source < sink) or less (source > sink) processed by the sinks. The degree of photochemical processing of CH_4_ thus produces strong changes in the Δ^12^CH_2_D_2_ signature, as shown by our simulations (Fig. 3).

The independently derived sink KIE values (~1.783 to 1.820 for ^12^CH_2_D_2_) in this study, calculated from the isotope budget mass balance using the firn air measurements, differ from previously published theoretical values (*18*, *27*) but are close to a published experimental result (*28*), which, however, has much larger uncertainties. The uncertainties regarding the derived KIEs are discussed in text S9 and fig. S9. Theoretical estimates of the clumped isotopic fractionation for CH_4_ + OH and CH_4_ + Cl reactions and their weighted temperature-dependent contributions to the total sink KIE may require reevaluation.

### CH_4_ changes in the future

Future atmospheric CH_4_ levels remain uncertain. Continued anthropogenic emissions and potential climate feedback from natural sources may lead to further increases in CH_4_ levels. Conversely, growing policy efforts, such as the Global Methane Pledge (*29*), which targets a 30% reduction in CH_4_ emissions by 2030 relative to 2020, could slow down or even reverse this trend. Achieving the Paris Agreement goals will require huge emission cuts and/or enhanced CH_4_ removal strategies. With this in mind, we performed simulations of future CH_4_ scenarios to examine how isotopic signatures respond to different future emission trends.

**Fig. 4.**
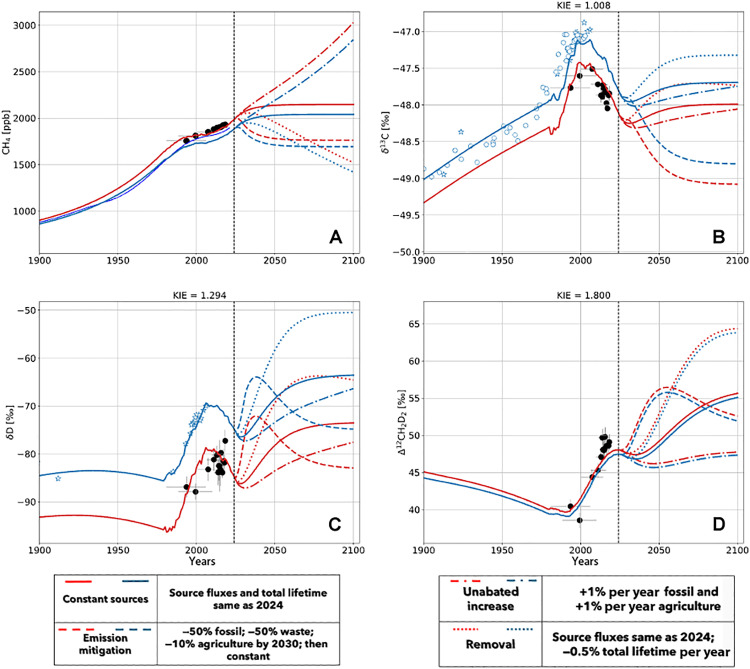
Results of long-term (1900–2100) atmospheric CH_4_ simulations of various future emission scenarios. (**A**) CH_4_ mole fraction, (**B**) δ^13^C, (**C**) δD, and (**D**) Δ^12^CH_2_D_2_. The vertical dashed black line represents the current year (2024). The black dots represent the firn air samples measured in this study, and each line (red for NH and blue for SH) corresponds to the results of model scenario tests as given in the legend.

Figure 4 shows the model results of CH_4_, δ^13^C, δD, and Δ^12^CH_2_D_2_ for four different scenarios, representing “constant sources,” “unabated increase,” “emission mitigation,” and “enhanced CH_4_ removal.” Additional scenarios using the Intergovernmental Panel on Climate Change’s (*30*) Shared Socioeconomic Pathways (SSP) are shown in fig. S11 (details in text S11). Different isotope signatures respond differently to future CH_4_ emission scenarios. δ^13^C reacts strongly to source composition changes because of the small sink fractionation (8‰ for δ^13^C). The relative shares of source categories remain largely unchanged in the three scenarios, “constant sources,” “increase,” and “removal,” resulting in minor temporal δ^13^C changes. The “mitigation” scenario, however, preferentially removes ^13^C-enriched sources (fossil and waste), thereby lowering δ^13^C values. The fossil source is also Deuterium enriched, suggesting that δD should decrease in the “mitigation” scenario, similar to δ^13^C. However, δD first increases in that scenario, as it is more sensitive to sink fractionation (294‰ for δD). Thus, the δ^13^C temporal evolution is dominated by source changes, while both source changes and the source-sink imbalance influence the evolution of δD.

Our modeling results demonstrate that the novel isotope tracer Δ^12^CH_2_D_2_ is particularly sensitive to source-sink imbalance. Under the “constant sources” scenario, it would take until the end of the century to approach a new equilibrium Δ^12^CH_2_D_2_ value of ~55‰. In the “mitigation” and “removal” scenarios, Δ^12^CH_2_D_2_ would further increase in the coming decades, driven by continued source-sink imbalance and the strong sink fractionation of 800‰. As in the past, Δ^12^CH_2_D_2_ will continue to reflect and integrate changes in the source-sink imbalance in the future. The isotopic tracers of CH_4_ will allow us to address the changes in key components of the CH_4_ budget: the sources (δ^13^C), the source-sink imbalance (Δ^12^CH_2_D_2_), and a mixed effect of both (δD).

## MATERIALS AND METHODS

The firn air samples used for this study were collected at the EastGRIP site in 2018. The firn air camp was located ~1 km east (upwind of the main wind direction) of the main EastGRIP camp to avoid possible contamination from activity at the camp. The firn air sampling is described in detail in (*15*) and in text S2. A total of 14 firn air samples was analyzed in this study. The cylinder pressures ranged from 56 to 111 bar, corresponding to 280 to 555 liters of air available for each analysis.

The mole fraction of CH_4_ in the cylinders was measured using a G2301 greenhouse gas analyzer (Picarro Inc.) at the Institute for Marine and Atmospheric research Utrecht (IMAU). The bulk isotopic composition (δ^13^C and δD) was measured directly from the cylinders using continuous-flow isotope-ratio mass spectrometry at IMAU (*31*). For the clumped isotopic composition measurements, samples were purified using a custom-built CH_4_ extraction system and analyzed using the Nu Panorama mass spectrometer at the University of Maryland following established procedures (*11*, *12*, *14*, *32*). The details of the extraction and measurement procedure are given in text S2.

For the interpretation of the results, we simulated CH_4_ transport and isotope fractionation in the EastGRIP firn column using the IGE-GIPSA forward firn air model, with site-specific diffusivity tuned to multitracer depth profiles (CH_4_, SF_6_, CFC-12, CFC-113, CH_3_CCl_3_, and HFC-134a). Modeled isotopologue profiles were forced with atmospheric CH_4_ and isotopic histories from the two-box CH_4_ isotopologue model and were used to correct measurements for gravitational settling and diffusive fractionation. The full model setup, age-distribution (Green’s function) calculations, and sensitivity tests are described in text S3.

## Supplementary Materials

### This PDF file includes:

Texts S1 to S11 Figs. S1 to S11 Tables S1 to S3 References
