# Supplementary material for: Anthropogenic perturbations to atmospheric methane reflected in Greenland firn air clumped isotope measurements
Source: Sci Adv. 2026 Jul 15;12(29):eaeb2203. doi: 10.1126/sciadv.aeb2203 (PMC13371891; doi:10.1126/sciadv.aeb2203)
Supplement: Supplementary file 1 — Texts S1 to S11 Figs. S1 to S11 Tables S1 to S3 References [file sciadv.aeb2203_sm.pdf]

Science Advances
AAAS

Supplementary Materials for

Anthropogenic perturbations to atmospheric methane reflected in Greenland firn air clumped isotope measurements

Malavika Sivan *et al.*

Corresponding author: Malavika Sivan, mals.sivan@gmail.com; Jiayang Sun, jiayangs@umd.edu; Thomas Röckmann, t.rockmann@uu.nl

Sci. Adv. **12**, eaeb2203 (2026)
DOI: 10.1126/sciadv.aeb2203

This PDF file includes:

Texts S1 to S11
Figs. S1 to S11
Tables S1 to S3
References

S1. Isotope notations and calculations

S1.1. δ and Δ notations

The bulk carbon and hydrogen isotopic compositions of CH_4, denoted as $\delta^{13}C$ and δD, are defined as:

$$\delta^{13}C_{sample} = \frac{R^{13C}_{sample}}{R^{13C}_{VPDB}} - 1 \qquad (S1)$$

$$\delta D_{sample} = \frac{R^{D}_{sample}}{R^{D}_{VSMOW}} - 1 \qquad (S2)$$

where, R^{13c}_{sample} and R^{D}_{sample} are the $^{13}C/^{12}C$ and D/H ratios of the sample, $R^{13c}_{VPDB} = 0.011180$ is the $^{13}C/^{12}C$ ratio of the international carbon isotope standard Vienna Pee Dee Belemnite, and $R^{D}_{VSMOW} = 0.00015576$ is the D/H ratio of the international hydrogen isotope standard Vienna Standard Mean Ocean Water.

The clumped isotopic composition of CH_4 in a sample is expressed as $\Delta^{13}CH_3D$ and $\Delta^{12}CH_2D_2$. It quantifies the deviation of the measured ratio of clumped isotopologues compared to the ratio that would be obtained if the heavy isotopes ^{13}C and D were distributed randomly across all isotopologues in the same sample:

$$\Delta^{13}\mathrm{CH_3D}_{sample} = \frac{R^{^{13}\mathrm{CH_3D}}_{sample}}{4*R^{^{13}\mathrm{C}}_{sample}*R^{\mathrm{D}}_{sample}} - 1 \qquad (S3)$$

$$\Delta^{12}\mathrm{CH_2D_2}_{sample} = \frac{R^{^{12}\mathrm{CH_2D_2}}_{sample}}{6*(R^{\mathrm{D}}_{sample})^2} - 1 \qquad (S4)$$

$R^{^{13}\mathrm{CH_3D}}_{sample}$ and $R^{^{12}\mathrm{CH_2D_2}}_{sample}$ are the isotopologue ratios of $^{13}CH_3D/^{12}CH_4$ and $^{12}CH_2D_2/^{12}CH_4$ of the sample. $R^{^{13}\mathrm{C}}_{sample}$ and R^{D}_{sample} are isotope ratios of $^{13}C/^{12}C$ and D/H of the sample. The denominators in the above formulae give the expected stochastic distribution of the heavier isotopes in a sample, where 4 and 6 are symmetry factors (*10*).

In practice, we use the following approximate equations to simplify the calculation of $\Delta^{13}CH_3D$ and $\Delta^{12}CH_2D_2$ (*11*):

$$\Delta^{13}\mathrm{CH_3D} = \frac{1+\delta^{13}\mathrm{CH_3D}}{(1+\delta^{13}\mathrm{CH_4})*(1+\delta^{12}\mathrm{CH_3D})} - 1 \qquad \text{(S5)}$$

$$\Delta^{12}\mathrm{CH_2D_2} = \frac{1+\delta^{12}\mathrm{CH_2D_2}}{(1+\delta^{12}\mathrm{CH_3D})^2} - 1 \qquad \text{(S6)}$$

The approximation is done by using the bulk isotopologue ratios, $^{13}CH_4/^{12}CH_4$ instead of $^{13}C/^{12}C$ and $^{12}CH_3D/^{12}CH_4$ instead of D/H. The uncertainties introduced by these approximations are much smaller than the analytical precision.

S1.2. Kinetic Isotope Effect and sink fractionation factors

The Kinetic Isotope Effect (KIE) is the rate constant ratio for chemical reactions involving different isotopologues. In our study, the KIEs are calculated as

$$KIE^i = \frac{k_{^{12}CH_4}}{k_i} \qquad \text{(S7)}$$

Where *i* is the isotopologue with heavy isotope substitution, i.e., $^{13}CH_4$, $^{12}CH_3D$, $^{13}CH_3D$, and $^{12}CH_2D_2$, and *k* is the rate constant of the specified isotopologue, for a specified reaction. For sink reactions (e.g., CH_4 + OH), the rate constants for methane isotopologues with heavy isotope substitutions are always smaller than that of $^{12}CH_4$, meaning the KIE^i is always greater than 1. The combined fractionation (KIE − 1) of the different sinks in the model is 8 ‰.

S2. Sampling and measurement methods

S2.1. Firn air sampling

The firn air samples used for this study were collected at the East Greenland Ice-Core Project (EastGRIP) site in 2018. The firn air camp was located approximately 1 km east (upwind of the main wind direction) of the main EastGRIP camp to avoid possible contamination from activity at the camp. The firn air sampling is described in detail in (*15*). In short, the firn hole was drilled to a certain depth, then the firn sampling assembly was inserted into the borehole. The assembly consisted of a 5 m long bladder and a set of three tubes, one to inflate the bladder and two to pump air from two different compartments below the bladder. The two compartments are separated by a stainless-steel disc with the same width as the borehole. First, the bladder is inflated via the fill tube so that it tightly seals the borehole, and the bottom of the borehole is isolated from the overlying atmosphere. The purge line pulls air from the first compartment below the bladder to further eliminate possible contamination from the bladder. The sample line pumps up the sample air from the lower second compartment. Continuous online CO_2 and CH_4 mole fraction measurements were performed to verify that no contamination with contemporary air occurred during the extraction procedure. After the contaminating air had been pumped away, firn air was collected into various containers for different analyses. The large volume air samples required for CH_4 clumped isotope analysis were compressed into 5L volume aluminium cylinders using two compressors in series, a metal bellows compressor and a RIX piston compressor (*33*). The RIX compressor can compress the air into the high-pressure cylinders up to a pressure of 140 bar, yielding up to 700 L of air for analysis. A total of 14 firn air samples were analysed in this study. The cylinder pressures ranged from 56 to 111 bar, corresponding to 280 to 555 L of air available for each analysis.

S2.2. Measurement methods for methane mole fraction and isotope analysis

After transport to the laboratory, the CH_4 mole fraction in the cylinders was measured using a G2301 greenhouse gas analyser (Picarro Inc.) at the Institute for Marine and Atmospheric Research (IMAU), Utrecht. The bulk isotopic composition ($\delta^{13}C$ and δD) was measured directly from the cylinders using a continuous flow isotope ratio mass spectrometry technique at IMAU (*31*).

For the clumped isotopic composition measurements, samples were shipped to the University of Maryland (UMD) for purification and analysis, following established procedures (*11, 12, 14, 32*). All remaining air in the cylinders was extracted and purified to isolate CH_4, ensuring complete recovery and minimising isotopic fractionation. Air was pulled through a water trap with dry silica beads at room temperature, followed by a large 6-loop U-trap filled with HayeSep DB Porous Polymer Adsorbent and cooled by liquid nitrogen, where CH_4, Kr, and remaining H_2O and CO_2 were trapped, but most N_2 and O_2 were pumped away. The large U-trap was then heated to 150 °C, and the trapped gases were pulled through a small U-trap filled with HayeSep DB cooled to liquid nitrogen temperature, further removing N_2 and O_2 while retaining CH_4. Ultra-pure N_2 from a cylinder was used to flush the sample from the large U-trap to the small U-trap to promote complete transfer of CH_4. The small U-trap was then heated to -120 °C using an ethanol slush bath and pumped for about 30 minutes, allowing further removal of N_2 and O_2 without CH_4 breaking through the HayeSep DB column. The remaining gases, including CH_4, Kr, and traces of N_2, O_2, H_2O, and CO_2, were transferred to the purification line.

The CH_4 in the sample was further purified using Gas Chromatography (GC) separation. Helium carrier gas was used to transfer the concentrated sample gas into a GC column packed with MolSieve 5A held at 56 °C. Under this condition, O_2, N_2, Kr, and CH_4 peaks eluted sequentially, so the CH_4 could be trapped selectively. Finally, the purified CH_4 was frozen into a glass sample vial with silica beads, sealed, and transferred to the Panorama mass spectrometer (*11*) for isotope and clumped isotopologue measurement.

The clumped isotopologue signals were measured using the Nu Panorama mass spectrometer at UMD. Panorama is a double-focusing, high-sensitivity, high-resolution gas-source electron-impact isotope-ratio mass spectrometer. It can achieve a mass resolving power (MRP) of ~55,000, sufficient to separate peaks of $^{13}CH_3D$ and $^{12}CH_2D_2$ from adjacent CH_4 adducts: $^{13}CH_5$ and $^{12}CH_4D$, which all have nominal mass 18. Each sample measurement is conducted using two measurement protocols. In the first run, $^{12}CH_3$ is measured on mass 15, $^{12}CH_4$ on mass 16, $^{13}CH_4$ on mass 17, and $^{13}CH_3D$ on mass 18. The second run, $^{12}CH_3$ on mass 15, $^{12}CH_4$ on mass 16, $^{12}CH_3D$ on mass 17, and $^{12}CH_2D_2$ on mass 18. The ion signals at masses 15, 16, and 17 are recorded by Faraday cups, while the ions at mass 18 are counted by an ion counter. Measurements are performed at balanced pressure and signal intensity between sample and reference pairs. The signal intensity of mass 16 is controlled at 10^{-10} Amps, and signal

intensities of mass 15, 17, and 18 are reported relative to mass 16. The detailed analytical process is described in (*11*).

The firn air samples (typically ~500 L, with some around 300 L) contained between 20 and 45 μmol of CH_4 (see Table S1 for more details), which is less than the ideal sample size for Panorama measurements (typically 70 μmol of CH_4). The limited sample quantity necessitates that measurements be completed within a shorter counting time. This results in higher measurement uncertainties compared to other samples analysed on the Panorama. For firn air samples, the 1-σ standard uncertainty of the mean (SE) for $\Delta^{13}CH_3D$ ranges from 0.2 to 0.4 ‰ for 5 to 7 hours of measurement time, and for $\Delta^{12}CH_2D_2$, it ranges from 1 to 1.5 ‰ for 12 to 20 hours of measurement time. The measurement results are given in table S1.

S3. Modelling trace gas transport in firn

Gas transport in firn is primarily driven by molecular diffusion, with additional mixing components in the upper layers and possibly deep firn (*34-37*). Given the long storage times of air in the firn layers, gravity induces the preferred sinking of heavier molecules, resulting in significant isotopic fractionation. Forward firn models use known or assumed temporal records of atmospheric composition as input and simulate trace gas profiles versus depth in firn. Here, we use IGE-GIPSA firn model (*15*), which showed excellent performance in a firn model inter-comparison study (*35*). A site-specific tuning is necessary to determine a firn diffusivity profile based on constraints provided by multiple gases for which both atmospheric trend and depth profile in firn are available (*35, 38*). Here, we use CH_4, SF_6, CFC-12, CFC-113, CH_3CCl_3, and HFC-134a in our reference simulation, using the same method as (*15*). The halocarbon measurements are produced by a separate group and will be presented in a dedicated study. Other combinations of reference gases for diffusivity tuning were tested (see below) and had a negligible impact on CH_4 clumped isotope results.

Inverse modelling is commonly used to reconstruct atmospheric trends from depth profiles of tracers measured in firn air samples. However, reconstruction of isotopic ratios requires specific model development, as they are not mass-conservative (*17*). Such specific development is not yet available for clumped isotope Δ values. Thus, we implemented a forward firn model approach using isotopic trends from the two-box model for atmospheric methane isotopologues (see S4). For the CH_4 mixing ratio that was used to convert isotopic δ

and Δ into mixing ratios of single isotopologues and back to isotopic δ and Δ, two trend scenarios were used: (i) a combination of ice core data and high-Northern latitudes atmospheric data including seasonal variations (for the atmospheric data) built as in (*35*) and intended to best fit Greenland firn data, and (ii) the Northern hemisphere model output of the 2-box atmospheric isotope forward model (see section S4) developed in this study. The firn model then calculates vertical profiles of the isotope composition of CH_4 in the firn layers, which can be compared to our measurements (Fig. 1). Similar approaches were used, for example, in (*34, 36*).

The atmospheric histories from the model simulations were plotted alongside measurement results as a function of time (Figs. 2-4, figs. S2, S6-S10), using the mean gas (*17*) of the corresponding firn air samples, after correcting for the effects of gravitational settling and diffusive fractionation as calculated by the firn air model. However, this introduces additional uncertainty due to the significant width of the gas age distribution in deep firn (fig. S1), represented as horizontal grey uncertainty bars in Figs. 2-4.

The age distribution of CH_4 in the firn column is evaluated using a Green's function approach: a Dirac delta function is used as an atmospheric forcing at the surface of the firn air model. This CH_4 pulse is then propagated into the firn, and the resulting matrix versus time and depth represents the age probability distribution function. Due to the effect of gravitational settling, the sum of all age probabilities at a given depth is slightly different from one and a small correction was applied to normalise the sum of probabilities to one at each depth. Fig. S1 illustrates the wide and asymmetrical CH_4 age distributions in firn air samples. For example, at the lowest measurement depth (64.2 m), the mode age (age of highest probability) is 9 years smaller than the mean age. The age uncertainties are calculated as ± 1σ equivalent age width, which represents the 70% probability age range (between 15% and 85% probability).

Several tests were performed to evaluate the robustness of our results.

Test 1: The large age widths shown in fig. S1 imply that firn air data do not constrain the early part of the atmospheric trends tightly. To evaluate the impact of the early atmospheric trends on the results, we tested modified scenarios before 1994 (the mean age of the deepest EGRIP sample, table S2). The best guess model simulations (black solid lines in left panels of fig. S2) were initialised in 1800 with an atmospheric CH_4 trend at high Northern latitudes evaluated as described in (*35*) (based on a combination of ice core data for the early trend and high Northern latitudes atmospheric data when available, black solid line in top right panel of fig. S2) and with the best guess isotopic ratios from the atmospheric model derived in this study (fig. S8). The impact of model initialisation of isotopic ratios with constant values and constant slopes before 1994 was evaluated (grey dashed and dash-dotted lines, respectively, in the right panels of fig. S2). This initial trend only affects the model results below 60 m depth and has a moderate impact on the isotope ratios.

Test 2: A final input scenario test compares the results obtained with the CH_4 trend intended to best represent the average NH trend (described in section S4, green solid lines in fig. S2), to those obtained with the best guess CH_4 trend for Greenland (black solid lines in fig. S2). Due to isotopic fractionation in firn, a change in the CH_4 mixing ratio trend induces changes in the isotopologue profiles in firn shown as the differences between black and green curves on the left panels of fig. S2. The best-guess high-latitudes trend includes seasonal variations in recent years, whereas the average NH trend represents annual and hemispheric means. EGRIP CH_4 mole fractions in firn in this study are somewhat underestimated when using the hemispheric CH_4 trend (green line), whereas for the isotopologues, only $\delta^{13}C$ is affected by this change in the input scenarios. We note that $\delta^{13}C$ in firn is strongly affected by the steep CH_4 trend and fractionation in firn, compared to its weak atmospheric variations (a few 0.1 ‰) (*17*). The wiggle in the top ~40m of the firn for the reference simulation reflects the effect of seasonal variations of CH_4.

The CH_4 mole fraction above Greenland prior to atmospheric measurements is not perfectly known (*39*). However, due to the short time coverage of EGRIP firn samples (Table S2), this affects our results less than the above tests. The main conclusion of the trend scenario tests is the weak sensitivity of $\Delta^{12}CH_2D_2$ to uncertainties in input scenarios.

On the right panels of fig. S2, original firn data (stars) are compared with data corrected for the effects of gravitational settling and diffusional fractionation (circles). Compared to atmospheric variations, the effect of firn processes is only important for $\delta^{13}C$. δD and clumped isotopologue signals remain largely unaffected. The high sensitivity of $\delta^{13}C$ to firn processes in recent decades, driven by the strong trend in CH_4 mole fraction and weak trend in $\delta^{13}C$, was described in detail in (*17*). In contrast, clumped isotopes Δ values exhibit a very weak sensitivity to fractionation in firn. This behaviour has been predicted theoretically (*40*) and confirmed experimentally (*41*) for molecular oxygen. The tests described below confirm this weak sensitivity to firn physics for clumped isotopes of methane at EGRIP.

Test 3: Only CH_4 was used to constrain the firn diffusivity profile instead of six different gases (red line in left panels of fig. S2). This results in a much less reliable diffusivity profile (*35, 38*). Again, only $\delta^{13}C$, which is highly sensitive to firn processes, is significantly affected (*16, 17*). Other combinations of reference gases for diffusivity tuning were tested (including some without using CH_4) and resulted in similarly negligible impact on clumped isotopes. The mean gas ages are also weakly affected and remain within the $\pm 1\sigma$ range provided in Table S2.

Test 4: Another important parameter in deep firn is the open/closed porosity ratio, which is poorly constrained (*15*). The blue lines in the left panels of fig. S2 illustrate the effect of using the modified Goujon open/closed porosity ratio described in (*15*). The results obtained are nearly the same as the reference simulations (black solid lines in the left panels of fig. S2).

Consistent with theoretical predictions (*40*), clumped isotopes exhibit a weak sensitivity to firn physics and fractionations in firn. We note that it implies that the relative proportions of molecular versus eddy diffusivity (*42*) will not affect the clumped isotope results.

S4. Two-box model for atmospheric methane isotopologues

S4.1. Model structure

The model divides the atmosphere into the Northern Hemisphere (NH) and Southern Hemisphere (SH) boxes. Using input data for emission rates and isotopic compositions of different CH_4 source sectors, mixing between the hemispheres, and the removal by the sinks, it simulates the temporal evolution of the different isotopologues of atmospheric CH_4 in NH and SH separately. (fig. S3). By design, a two-box model assumes that the CH_4 mole fraction in each hemisphere is well-mixed. It should be noted that this assumption is not accurate. Specifically, the measurements of CH_4 mole fraction and isotopic composition in the firn-trapped air on the Greenland ice sheet are not representative of mean NH values. We apply corrections for intra-hemispheric gradients to account for the differences between the measured values at polar latitude and the typical average hemispheric values (*43, 44*). The model assumes an interhemispheric exchange time of 0.75 years, constrained by independent model studies with SF_6 (*45*).

The model simulates individual isotopologue mole fractions of $^{12}CH_4$, $^{13}CH_4$, $^{12}CH_3D$, $^{13}CH_3D$ and $^{12}CH_2D_2$. Thus, for each source category, the total flux is expressed as individual isotopologue fluxes using the isotopic source signatures. Then, the isotopologues from all sources are added, transported, and removed (by applying the KIE to simulate isotopologue-specific removal rate coefficients) in the model. The δ and Δ values are then calculated again from the isotopologue abundances. The source categories of CH_4 are fossil, pyrogenic, wetlands, waste, and agriculture (see section S5), while the sinks include chemical reactions with OH and Cl radicals in the troposphere, stratosphere, and soil sinks.

The model defines the relationship of the observed mole fractions y (of each species) to the state vector x (emission sources in Tg/y) such as $y = F(x) \pm \sigma_\epsilon$, where F is the model operator and σ_ϵ^2 is the observational error variance, defined as the sum of observations and model uncertainties.

S4.3. Source-specific input scenarios

The annual input source fluxes are constrained by ice core measurements from the Law Dome, West Antarctic Ice Sheet (WAIS) ice core (*4, 16, 21*) and direct atmospheric measurements at northern high latitudes (*46*). The timeline was divided into five periods as shown in fig. S4. The periods TS1 (990-1460), TS2 (1461-1575) and TS3 (1589-1730) correspond to those defined by (*21*). The source mixes derived for these time slices from ice core measurements were smoothly interpolated between TS3 and TS5 to obtain annual emissions per category in TS4. A constant lifetime of 7.6 years was used in (*21*). This is much lower than suggested in recent studies (~9 years) (*47*). In our model, the yearly optimised lifetime output of the inverse model (*20*) optimised using methane mole fraction and bulk isotopes (see below) was used for the years 1980 to 2024, and a constant lifetime of 9.2 years for SH and 8.9 years for NH was used from 1000 to 1979 for consistency. Therefore, in our model, the source fluxes for TS1, TS2 and TS3 used in (*21*) were scaled accordingly to fit the atmospheric CH_4 budget for that period.

Another important distinction between the emission fluxes used in this study and in (*21*) is the contribution of pyrogenic vs geological fossil emissions. In (*21*), pyrogenic emissions outweigh fossil emissions in the period 990-1730. However, this doesn't reproduce the measured δD values of ice core samples collected from WAIS Divide. Therefore, to match all the available ice core and firn data, we performed simulations with more geological fossil emissions in the pre-industrial period, as shown in fig. S4.

In the period TS5 (1980-2024) optimised fluxes for each source, obtained from an inverse modelling study (*20*), were used. These fluxes are constrained by the bulk carbon and hydrogen isotopic composition measurements from high-latitude sites in both hemispheres over the past 30 years. The period TS4 (1730-1980) is the intermediate period that lacks measurement constraints. We interpolate the annual fluxes with a smooth exponential evolution for all source categories from 1730 to 1980 that aligns with the known CH_4 mole fraction, δ^{13}C and δD changes (*21*). Both the inverse and the forward models have the same model framework.

The details of the bulk isotope inverse model are described in (*20*). Briefly, the prior flux dataset was obtained from a combination of data from several inventories: Emissions Database for Global Atmospheric Research (EDGAR), Atmospheric Chemistry and Climate Model

Intercomparison (ACCMIP)/MACC and CityZen projects (MACCity), Global Fire Assimilation System (GFAS), Lund–Potsdam–Jena Wald Schnee und Landscaft version (LPJ-wsl), and Trends in Precipitation and its Predictability (TIPP). The state vector x of emission rates was optimised by minimising the cost function: $J(x) = (x - x_{prior})^2/\sigma_a^2 + (y - F(x))^2/\sigma_\epsilon^2$, with x_{prior} the a-priori emission rates in Tg/y, based on bottom-up inventories, and their variance σ_a^2. The inversion was performed to fit the atmospheric CH_4 mole fractions and bulk carbon and hydrogen isotopic compositions. These optimised inverted source fluxes and lifetimes outputs (fig. S5) were used as the input for the forward model in this study that incorporates the isotopologues $^{13}CH_3D$ and $^{12}CH_2D_2$ to study the evolution of atmospheric methane clumped isotopologues.

S5. Definition of source categories

CH_4 sources are classified into 5 categories: fossil, pyrogenic, wetlands, waste, and agriculture. Different inventories and studies classify CH_4 emissions differently, so clarification is necessary. Generally, there are two overlapping classification methods: process-based and activity-based. The process-based method categorises CH_4 as microbial, thermogenic, or abiotic (pyrogenic). The activity-based method classifies CH_4 emissions as anthropogenic or natural and may further specify them by type of activity, such as fossil fuel-related, agriculture, waste, biomass burning, wetlands, waters, geological sources, oceans, termites, wild animals, and permafrost. Each specific CH_4 source category can be further subdivided. For example, fossil fuel-related emissions may come from oil, natural gas, and coal and in inventories, they are further categorised into production, gathering, processing, transmission and storage, local distribution, oil refining, and transportation. Global CH_4 emission inventories typically use the activity-based classification method, for example, the Global Carbon Project (GCP), EDGAR, and the Intergovernmental Panel on Climate Change (IPCC) Assessment Report (IPCC AR6). GCP focuses specifically on CH_4, whereas IPCC and EDGAR cover all greenhouse gases, leading to slight differences in classifications but generally consistent categories. Forward models often use a combination of classifications from large databases.

We employed a mixed definition approach where fossil, wetlands, waste, and agricultural sources follow the definitions in GCP, while the pyrogenic category represents the remaining sources. When using EDGAR and IPCC datasets, re-categorisation is necessary. The following rules were adopted:

We classify the IPCC-defined category "1 Energy" as *fossil*, although "1A Fuel Combustion Activities" might fit under *pyrogenic*. IPCC categories "2 Industrial Processes and Product Use," "3C1 Biomass Burning," and "5 Other" are categorised as *pyrogenic*, although some microbial emissions (e.g., termites, oceans) are also included in "5 Other". Categories "3B4 Wetlands," "3B1 Forest Land," and "3B3 Grassland," along with any residual fluxes, are categorized under *wetlands*. This adjustment is necessary because IPCC and EDGAR focus on anthropogenic emissions. "3A2 Manure Management" and "4 Waste" are classified as *waste*, while "3A1 Enteric Fermentation" and "3B2 Cropland" (mainly rice) fall under *agriculture*. For detailed definitions of specific sources, such as what type of land can be defined as wetlands, we refer to the IPCC guidelines (*48, 49*), which are beyond our discussion scope.

This mixed definition approach is necessary because our model involves isotopes. Only under the process-based classification do different CH_4 source categories exhibit distinct isotopic signatures. On the other hand, the isotope signatures in the model need to be flux-weighted, and reliable flux constraints are primarily available under the activity-based classification framework.

S6. Defining the isotopic composition of the source categories

Based on our current understanding of fossil, pyrogenic, wetlands, waste, and agriculture sources, we assigned $\delta^{13}C$, δD, $\Delta^{13}CH_3D$ and $\Delta^{12}CH_2D_2$ values to each source. The $\delta^{13}C$ and δD values were differentiated between the NH and SH to account for hemispheric difference, whereas globally uniform values were applied for $\Delta^{13}CH_3D$ and $\Delta^{12}CH_2D_2$ due to limited availability of regional-specific data.

S6.1. Bulk isotopic compositions

The bulk isotopic signatures, $\delta^{13}C$ and δD of CH_4, were optimised using the inverse model described above, using prior values for different source categories that are assigned as averages derived from previously published papers and global databases (*20*). A large number of sensitivity tests were run with the inverse model (*20*), varying input parameters such as the source isotope signatures obtained from literature, and evaluating the posterior results. Based on these simulations, source signatures were revised to optimise the model output, i.e., to improve the match between posterior outputs and atmospheric observations. The optimised hemisphere-specific source signatures are adopted in this work in the 2-box atmospheric forward model and are given in table S3.

S6.2. Clumped isotopic compositions

For the clumped isotopologue signatures of each source, we evaluated currently published CH_4 clumped isotopologue measurements (the data used are available in the supplementary file with information on the publications they are taken from) (*10, 11, 50-80*). Samples from oil and natural gas well sites, hydrothermal vents, seeps, steam vents, residential natural gas, and gas hydrates are categorised as fossil-related, regardless of whether clumped isotopologues indicate microbial or thermogenic origins or microbial alteration. Samples from vehicle exhaust, biomass burning, lab pyrolysis experiments, and lab coal heating experiments are considered pyrogenic. Natural samples from ponds, swamps, permafrost, lake and ocean sediments, lake bubble ebullition, wetlands, and derived values from wetland air plumes, along with lab pure-culture methanogenesis, aerobic oxidation of methane (AOM), and anaerobic oxidation of CH_4 (AeOM), are categorised as wetlands. Samples from cow rumen and rice

paddies are categorised as agriculture. Landfill compositions derived from landfill plume air samples are used for waste. Measurements for each category were averaged or weighted.

Due to the disproportionate number of samples from synthetic laboratory experiments in the pyrogenic category, averages for natural and lab samples were calculated separately and then averaged at an 80:20 ratio. This method also applies to the wetland category, with a 75:25 ratio for natural to lab samples. A high number of samples in the wetland category may be affected by aerobic and anaerobic oxidation. These are typically low in mole fraction and trapped deep in the sediment, so they likely do not represent the true emission signals from wetlands. Therefore, we used a weighted ratio of methanogenesis-produced CH_4 to oxidised-CH_4 in proportions 85:15. The agriculture and waste categories had few measurements, possibly lacking representativeness. While these classifications, weightings, and final values are limited, they represent our best understanding of CH_4 clumped isotopologues.

Some studies only measured Δ_{18}, which is a combination of $^{13}CH_3D$ and $^{12}CH_2D_2$, due to the limitations of the mass spectrometer resolution. These Δ_{18} values were approximated as $\Delta^{13}CH_3D$, because $^{12}CH_2D_2$ abundance is two orders of magnitude lower than $^{13}CH_3D$, and its influence should be negligible. Additionally, some studies using laser spectrometers only measured $\Delta^{13}CH_3D$ and lack $\Delta^{12}CH_2D_2$ values. Systematic differences may exist between different labs and instruments, but these variations are not considered in this study.

Table S3 shows the final averaged clumped isotopologue signatures of each source category. Fossil CH_4 has $\Delta^{13}CH_3D$ = 3.6 ‰ and $\Delta^{12}CH_2D_2$ = 7.6 ‰, slightly below the thermodynamic equilibrium line. This is because fossil methane includes both thermogenic and microbial methane (e.g., (*9*)). Pyrogenic methane is assigned values of $\Delta^{13}CH_3D$ = 1.2 ‰ and $\Delta^{12}CH_2D_2$ = -9.5 ‰, consistent with biomass burning and lab pyrolysis measurements, which have shown slightly anti-clumping signals in $\Delta^{12}CH_2D_2$ (*11, 46*). Wetland CH_4 is assigned $\Delta^{13}CH_3D$ = 1.7 ‰ and $\Delta^{12}CH_2D_2$ = -36.0 ‰, agriculture CH_4 is assigned $\Delta^{13}CH_3D$ = -0.2 ‰ and $\Delta^{12}CH_2D_2$ = -39.8 ‰, and waste CH_4 is assigned $\Delta^{13}CH_3D$ = 2.3 ‰ and $\Delta^{12}CH_2D_2$ = -27.2 ‰. These results align with the understanding that microbial methanogenesis produces strong anti-clumping in $\Delta^{12}CH_2D_2$ and that waste CH_4, having undergone more oxidation, shows less anti-clumping. The total source mixture for the year 2025 using the above signatures is around 2.2 ‰ (SH) and 3.2 ‰ (NH) for $\Delta^{13}CH_3D$ and -29.4 ‰ (SH) and -13.1 ‰ (NH) for $\Delta^{12}CH_2D_2$

S7. Effect of source changes on atmospheric methane mole fraction and isotope trends in 1980 - 2024

To understand the reason for the measured variability in isotopic signatures of firn samples in this study, the sensitivity of the CH_4 mole fraction and all the isotopologues to the contributions from individual source categories was tested by keeping one source constant over time (as in 1980) and using the optimised fluxes for others. This was done for all five source categories, and the model was run from 1980-2024. The optimised lifetimes were used in all calculations. Fig. S6 shows that these tests drastically change the CH_4 mole fraction in the atmosphere, which is expected if changes in one source category are excluded. The different scenarios also lead to clear changes in $\delta^{13}C$, especially between the constant fossil and other scenarios. The changes in δD are much smaller but still significant and distinguishable with the presently available analytical precision. However, the difference in $\Delta^{13}CH_3D$ and $\Delta^{12}CH_2D_2$ between these different scenarios is very small and within the current achievable measurement uncertainty.

In a second sensitivity study, we still kept the flux from one source category constant but adjusted the other categories correspondingly, which resulted in simulation results where the atmospheric CH_4 mole fraction was not changed. Fig S7 shows that the bulk isotopes, $\delta^{13}C$ and δD, react the most to these changes, and the clumped isotope anomalies show minor changes, and the differences are within the measurement uncertainty. Both these tests show that the measured 10 ‰ change in $\Delta^{12}CH_2D_2$ cannot be explained solely by the change in the individual source fluxes from 1980-2024.

S8. Long-term model output

The long-term model output covering 1300-2024 is shown in fig. S8.

S9. Constraining the KIEs in the CH_4 sink reactions

As mentioned in the main text, the derived sink KIEs are directly impacted by the assigned source isotopic signatures, to fit with atmospheric observations. By varying the source isotopic compositions within a reasonable range, it is possible to adjust the KIEs such that the box model still produces simulations that are consistent with the new firn air observations (fig. S9A and S9C). KIEs and source isotopic signatures have linear relationships (fig. S9B and S9D). The uncertainty in the removal reaction KIEs of CH_4 can be evaluated by the uncertainties in the assigned source isotopic signatures of the total source.

In order to assess this uncertainty, we varied the source isotopic signatures of $\Delta^{13}CH_3D$ and $\Delta^{12}CH_2D_2$ for the different source categories over a range of reasonable values and optimised the KIEs for each assumed source signature (fig. S9). The reasonable range for $\Delta^{12}CH_2D_2$ of the source mixture (-21 ‰ to -4 ‰, yellow horizontal band in fig. S9D) is calculated using the most common reported range of compositions measured for the microbial samples to date, -50 ‰ to -20 ‰ for $\Delta^{12}CH_2D_2$. For $\Delta^{13}CH_3D$, the reasonable range for the total source is constrained to be 1.8 ‰ to 3.5 ‰ (yellow horizontal band in fig. S9B), which is calculated assuming that sources with non-equilibrium signatures have $\Delta^{13}CH_3D$ values to vary between 0 and 2 ‰.

Based on the given range of source isotopic compositions, we constrained the sink KIEs to the range of 1.303 to 1.305 for $^{13}CH_3D$, and 1.783 to 1.820 for $^{12}CH_2D_2$. Particularly for the KIE of $^{12}CH_2D_2$, the derived uncertainty range of ±0.02 is remarkably narrow, representing a significant improvement in the estimation of this parameter. Previous estimates relied on first-principles calculations or challenging experimental measurements, but the reported values have shown large inconsistencies. Theoretical calculations tend to yield higher values around 1.88 (represented by light blue circles in fig. S9), whereas some experimental studies have reported lower values around 1.73 or even lower (represented by dark blue circles in fig. S9).

S10. Model tests with different past emission scenarios

Model test results under different past emission scenarios are shown in fig. S10.

S11. Future Emission Scenarios

We evaluate the CH_4 mole fraction and the isotopic composition for several future emission scenarios. The *constant sources* scenario kept the flux of each source between 2024 and 2100 at the 2024 levels (solid red lines in Fig. 4).

The *emission mitigation* scenario (dashed red lines in Fig. 4) shows the evolution of CH_4 mole fraction and isotope signatures for a 50 % reduction of fossil fuel emissions and 10 % of agriculture (including ruminants) emissions by 2030, assuming a linear decrease per year from 2024 to 2030. This scenario aligns with what the Global Methane Pledge (*29*) promotes. In this case, we assume that the fluxes stay constant after 2030.

The *removal* scenario (solid purple (NH) and solid olive (SH) lines in Fig. 4) brings down the mole fraction to 1500 ppb in 2100 by keeping the source fluxes constant as of 2024, and by decreasing the total lifetime of atmospheric CH_4 by 0.5% each year.

The *unabated increase* scenario (dashed-dot red lines in Fig. 4) assumes an increase of the main anthropogenic (fossil and agriculture) sources by 1 % each year from 2024 till 2100. In this scenario, the CH_4 mole fraction reached about 3000 ppb in 2100.

We also used the IPCC-defined Representative Concentration Pathways (RCPs), with future emission fluxes from the IPCC AR6 Scenarios Database (*30, 81, 82*). We selected five SSPx-Baseline scenarios run by the IMAGE3.0.1 (*83*) model, which provides methane emissions specific to source categories. SSP represents Shared Socio-Economic Pathways, with higher numbers indicating higher target radiative forcing levels by 2100. To align with our model, we reclassified IPCC's future emission inventories according to our source categories, as explained in section S5. In addition, the SSPs only provide emission estimates at five-year intervals and differ slightly from the optimized emissions in our two-box model in both magnitude and trend. Moreover, the emission estimates from different SSPs are only consistent before 2015, while values from 2020 onward are based on projections. Therefore, adjustments were necessary. The emission fluxes from the SSPs were first scaled (i.e., multiplied by a correction factor across all 5-years) to match our model's 2015 emission fluxes for each source. Subsequently, cubic interpolation was applied to convert the five-year interval flux estimates into annual values. The interpolated SSP emissions were then offset (i.e., a uniform value was added or subtracted

across all years) to match the 2024 emission fluxes in our two-box atmospheric model, ensuring a consistent starting point for that year. Finally, each category's total emissions were divided into SH and NH based on the 2015 NH/SH emission ratio. This process ensures that RCP inputs fit the observational data in 2024 while retaining the temporal evolution of the RCP estimates for future emission trends. We keep the wetlands emissions constant from 2024 to 2100 in all the scenarios. The evolution of CH_4 mole fraction, $\delta^{13}C$, δD, $\Delta^{13}CH_3D$ and $\Delta^{12}CH_2D_2$ resulting from the emission time series of all the SSP scenarios are shown in Fig. S11.

Figures and captions

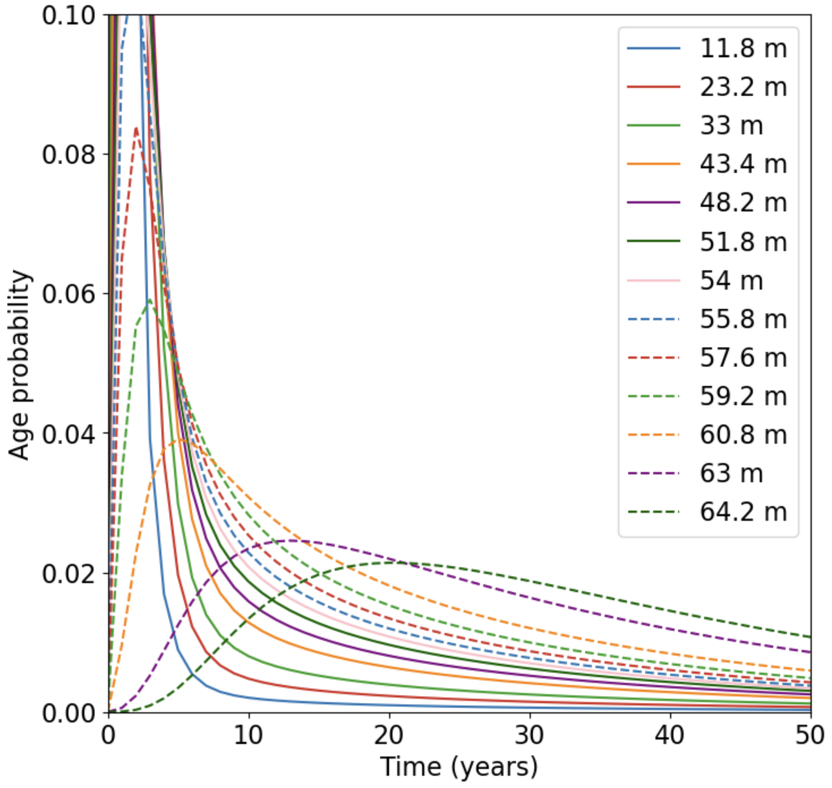

Fig. S1. Modelled methane age distribution for the firn air samples collected at the EastGRIP site. Each line represents the age probability distribution at the sampling depth given in the legend. The x-axis shows the time (in years) before 2018 (the firn drilling year).

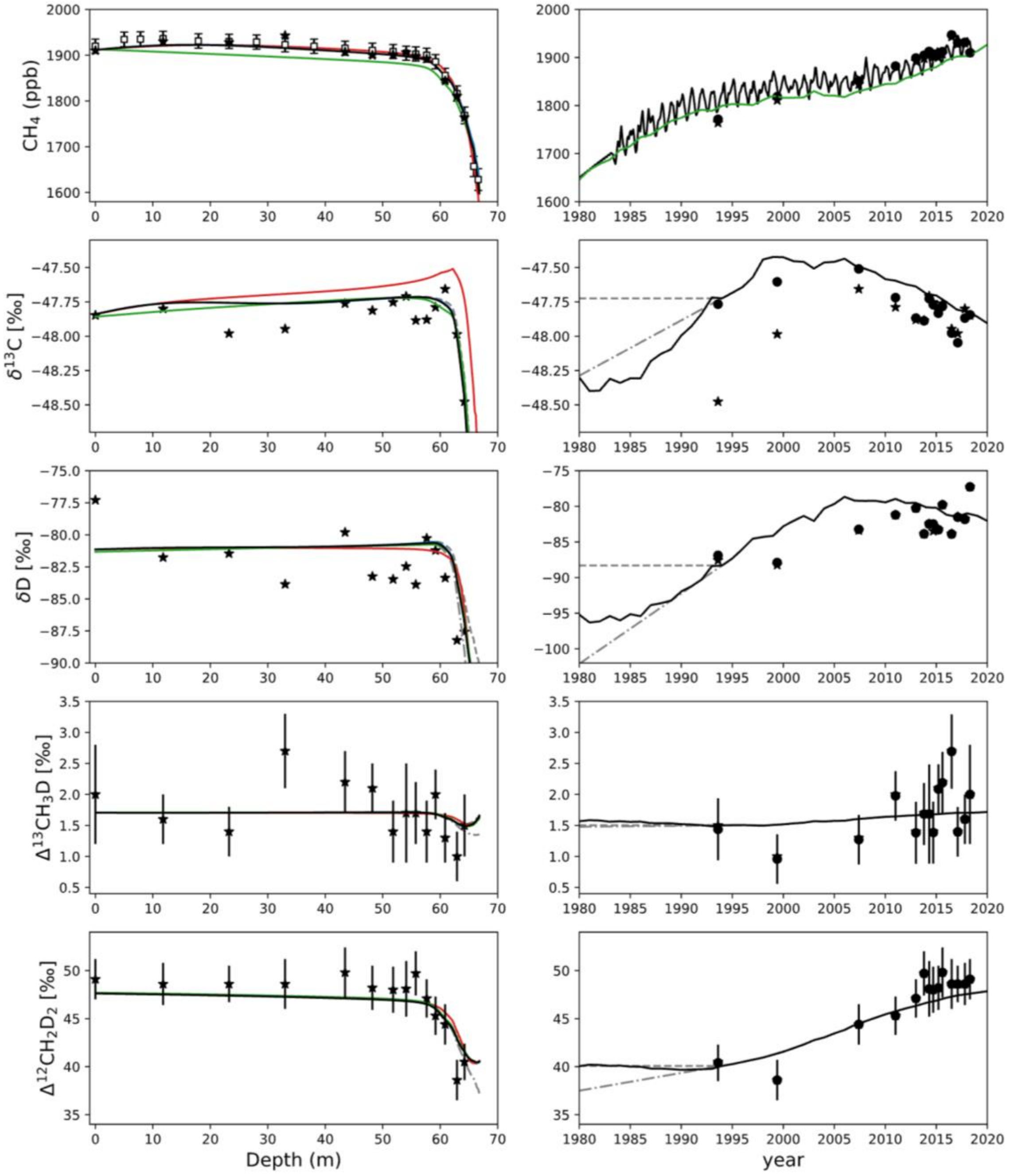

Fig. S2: Firn model sensitivity tests. Left panels: Comparison of firn model results (lines) with firn measurements (symbols) as a function of depth. For CH_4, stars show measurements from this study (Utrecht University), while open squares with uncertainty bars represent CH_4 mole fractions measured at IGE (from the same firn hole, published in (*15*)). Reference simulations using the CH_4 atmospheric trend based on high Northern latitudes data are shown as black dashed lines. Sensitivity test results are indicated by grey dashed/dash-dotted lines and colored solid lines (green, red, and blue; see Section S3 for details). **Right panels:** Comparison of modelled atmospheric trends (lines) with firn data plotted at their mean gas age. The black

solid line in the top panel represents the CH_4 high-latitude atmospheric trend, whereas the green solid line depicts the atmospheric two-box model output (details in Section S4). Stars represent measurements from this study (table S1), and solid circles show data corrected for gravitational settling and diffusional fractionation (table S2). For $\Delta^{13}CH_3D$ and $\Delta^{12}CH_2D_2$, the data corrected from firn fractionation are indistinguishable from the raw data. Reference isotopic scenarios from the two-box atmospheric model (see section S4) are shown as black solid lines. Isotope ratio trends with constant value or constant slope before 1994 are shown as grey dashed and dash-dotted lines, respectively.

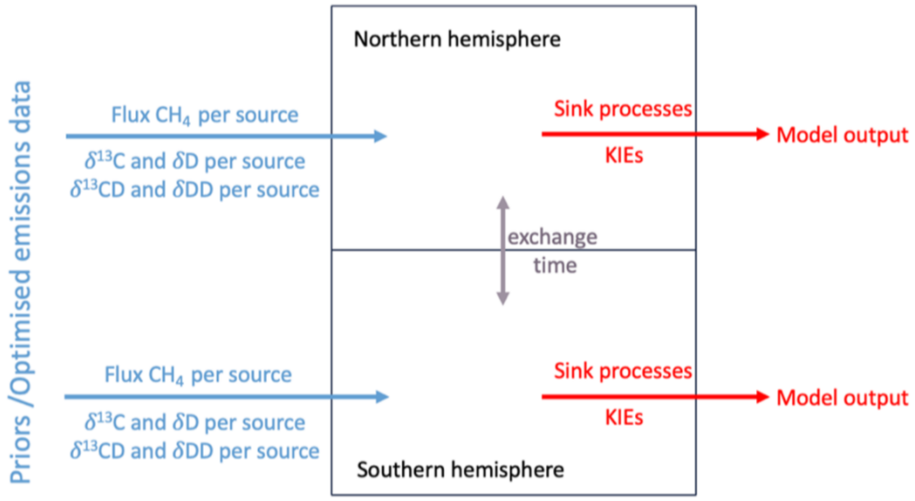

Fig. S3. Schematic of the two-box atmosphere model used in this study.

The two-box clumped isotope forward model, developed for this work, uses emission fluxes of each source category, isotopic compositions of each source category, and atmospheric lifetimes of each isotopologue as input parameters. The outputs are atmospheric CH_4 mole fractions and isotopic compositions. Detailed model structures are described in section S4.1. Input scenarios of emission fluxes of each source category are described in section S4.2. Definitions of source categories are given in section S5. Determinations of input clumped isotopologue compositions of each source category are introduced in section S6.

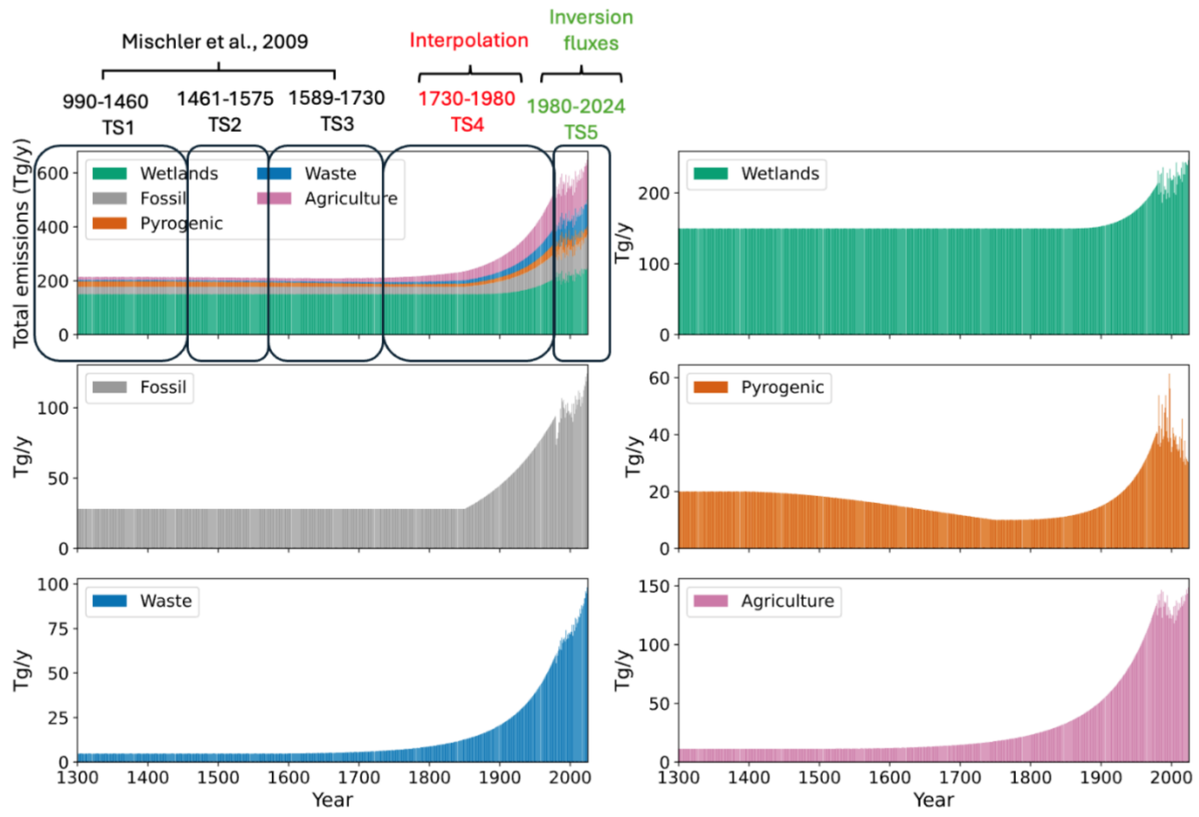

Fig. S4. The methane source fluxes (in Tg/year) per source category for the years 1300 to 2024. The timeline is divided into 5 periods (TS1 – TS5), and the source fluxes were assigned as described in section S5.2.

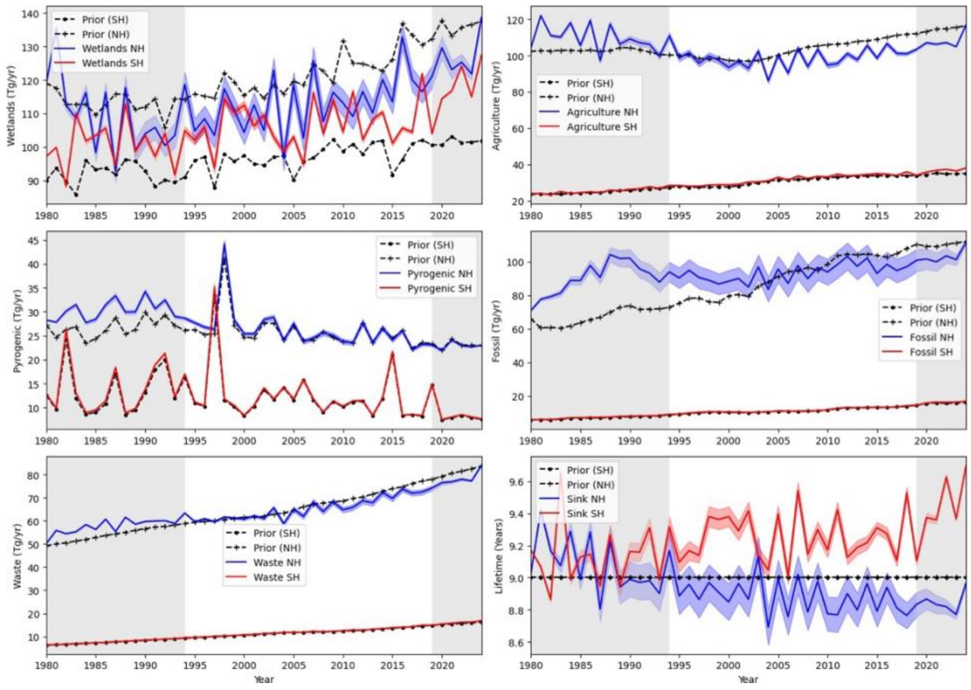

Fig. S5: Annual prior and posterior emission fluxes per source sector and lifetime. Different panels represent CH_4 fluxes for each source category as given on the Y-axis. Prior emission rates from EDGAR, ACCMIP/MACCity, GFAS, LPJ-wsl and TIPP are shown as black symbols, SH: dots, NH: plus symbols. Posterior emission rates are plotted in blue for NH and red for SH. Red and blue shaded regions are 3-σ uncertainty bands. Grey shaded regions are spin-up and spin-down periods of the inverse model (*20*).

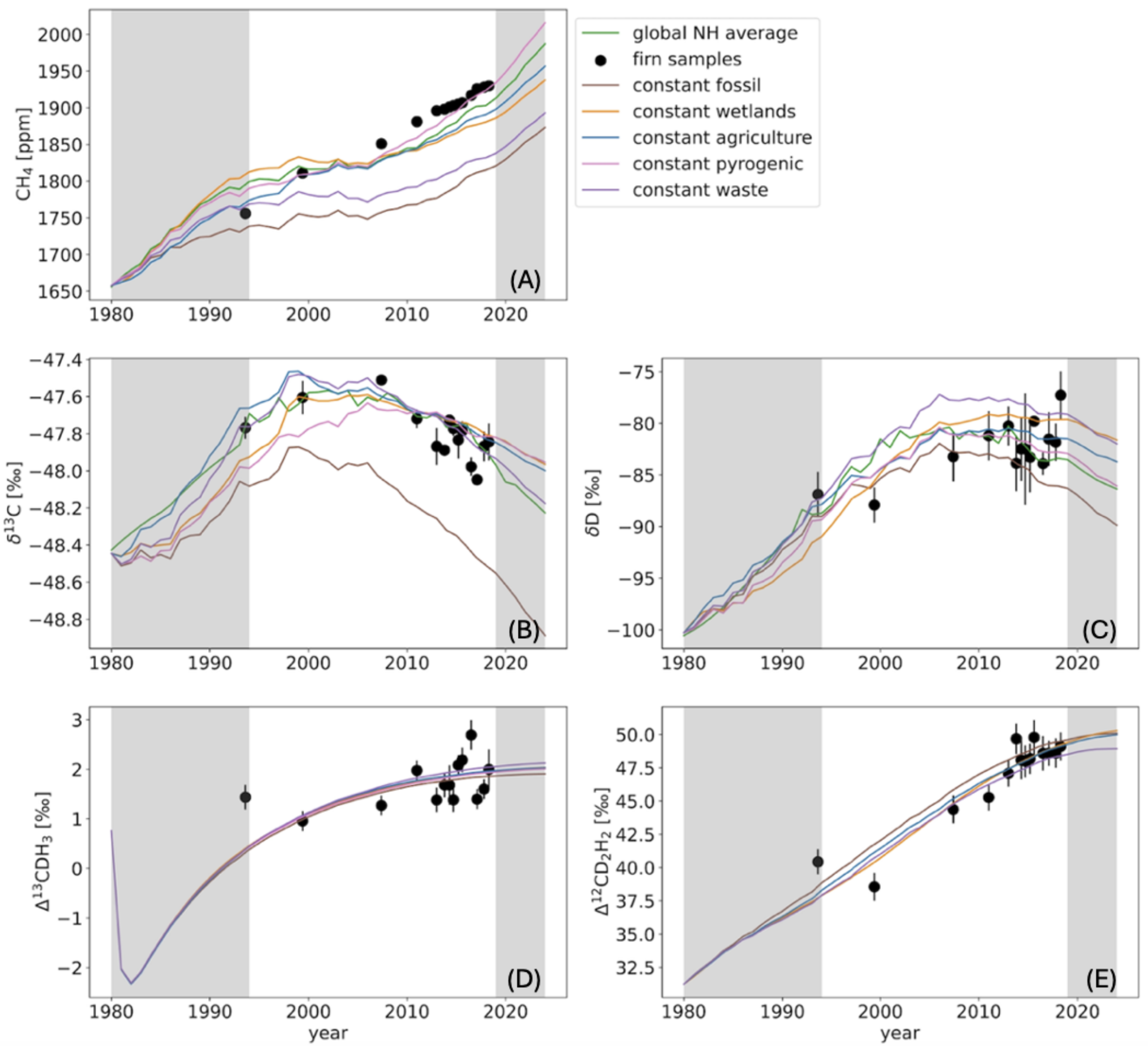

Fig S6. Results of sensitivity tests of atmospheric CH_4 mole fraction and isotopic signature to individual source contributions. (A) CH_4 mole fraction, **(B)** $\delta^{13}C$, **(C)** δD, **(D)** $\Delta^{13}CH_3D$, and **(E)** $\Delta^{12}CH_2D_2$.The colours represent the scenarios when one of the sources is kept constant from 1980-2024, as given in the legend. The solid black circles represent the firn samples measured in this study.

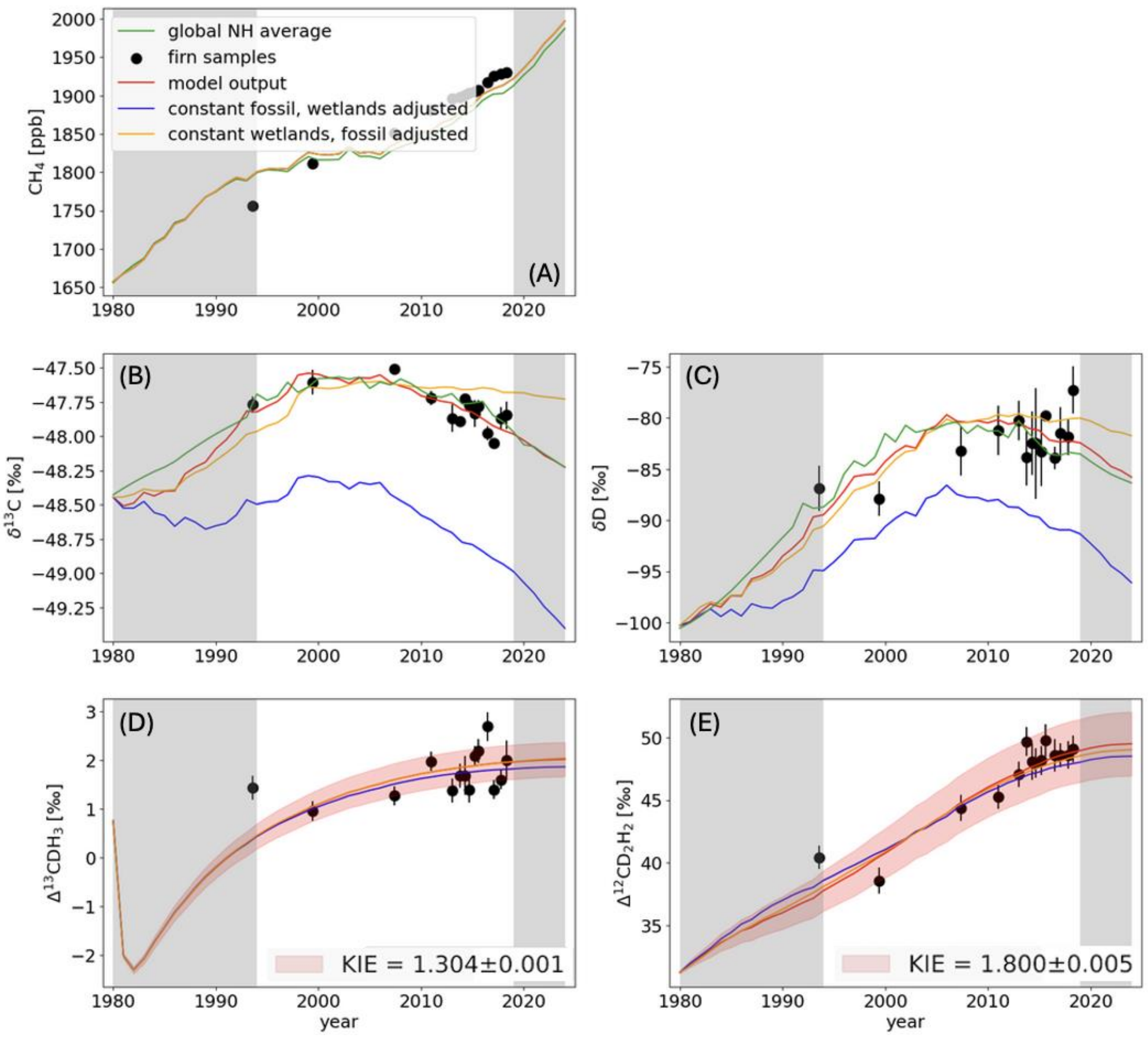

Fig S7. Results of model simulations using modified emission scenarios. (A) CH_4 mole fraction, **(B)** $\delta^{13}C$, **(C)** δD, **(D)** $\Delta^{13}CH_3D$, and **(E)** $\Delta^{12}CH_2D_2$. The blue solid lines represent the scenario with constant fossil emissions while adjusting wetland emissions to match observed atmospheric methane mole fraction, and the yellow lines represent the scenario with constant wetland emissions while adjusting fossil emissions to match observed atmospheric methane mole fraction when one of the sources is kept constant from 1980 to 2024, as given in the legend. The solid black circles represent the firn samples measured in this study.

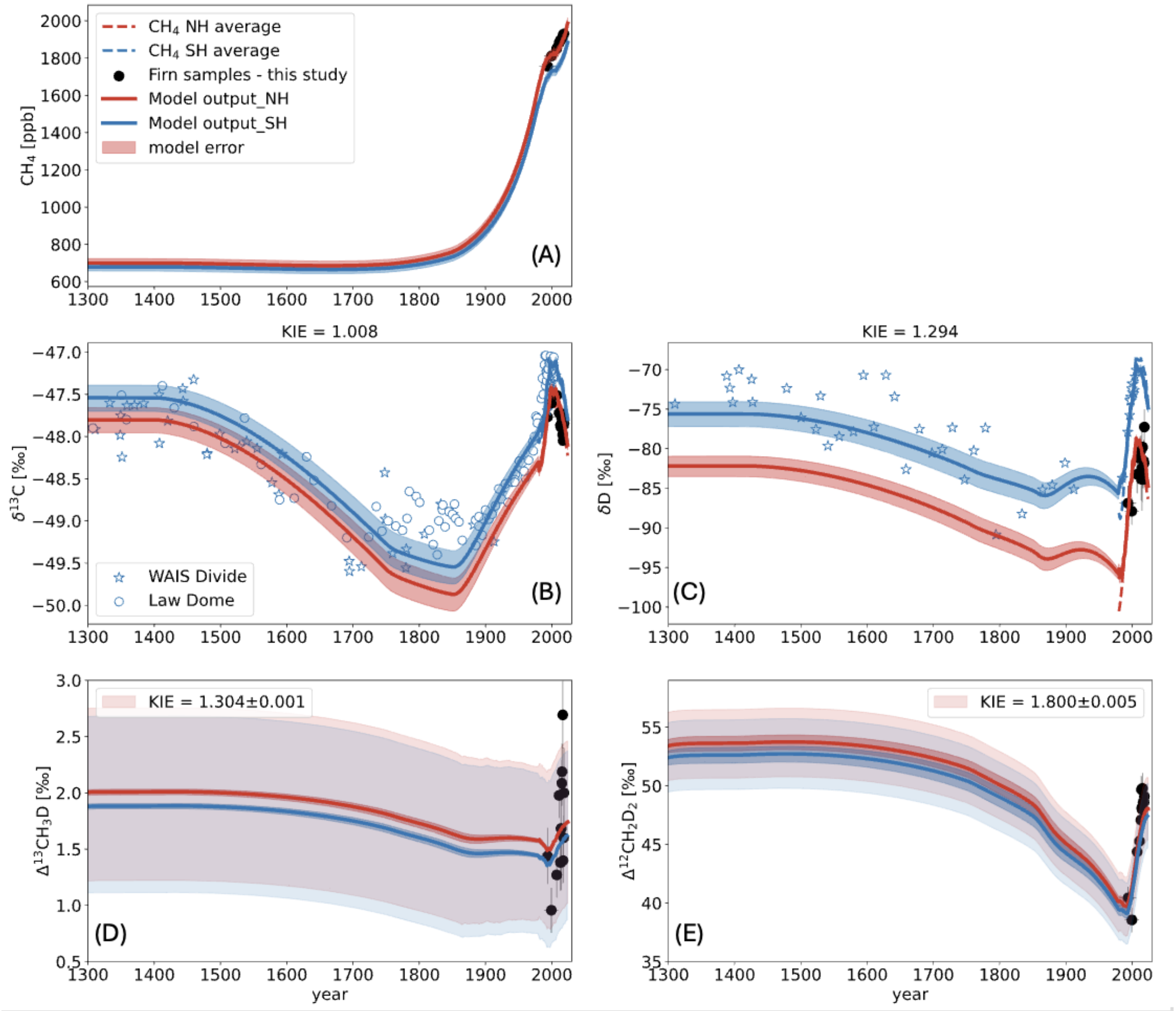

Fig. S8. Measured and modelled long-term temporal evolution of atmospheric CH_4 mole fractions and isotopic signatures from 1300-2024. (A) CH_4 mole fraction, **(B)** $\delta^{13}C$, **(C)** δD, **(D)** $\Delta^{13}CH_3D$, and **(E)** $\Delta^{12}CH_2D_2$. Measurements from this study are shown in black circles. Measurements from previous studies for the SH (*8, 21*) are shown in blue circles and stars. Model outputs of the two-box forward model (this study) from 1300 to 2024 using the fluxes (described in S4.2 and shown in fig. S4) are shown in red line for NH and blue line for SH. The sink reaction KIEs for $^{13}CH_4$, $^{12}CH_3D$, $^{13}CH_3D$, and $^{12}CH_2D_2$ are given as legends in (B)-(E). The red and blue shaded areas in (D) and (E) show the sensitivity of the KIEs used (±0.001 for $\Delta^{13}CH_3D$ and ±0.005 for $\Delta^{12}CH_2D_2$) for NH and SH, respectively.

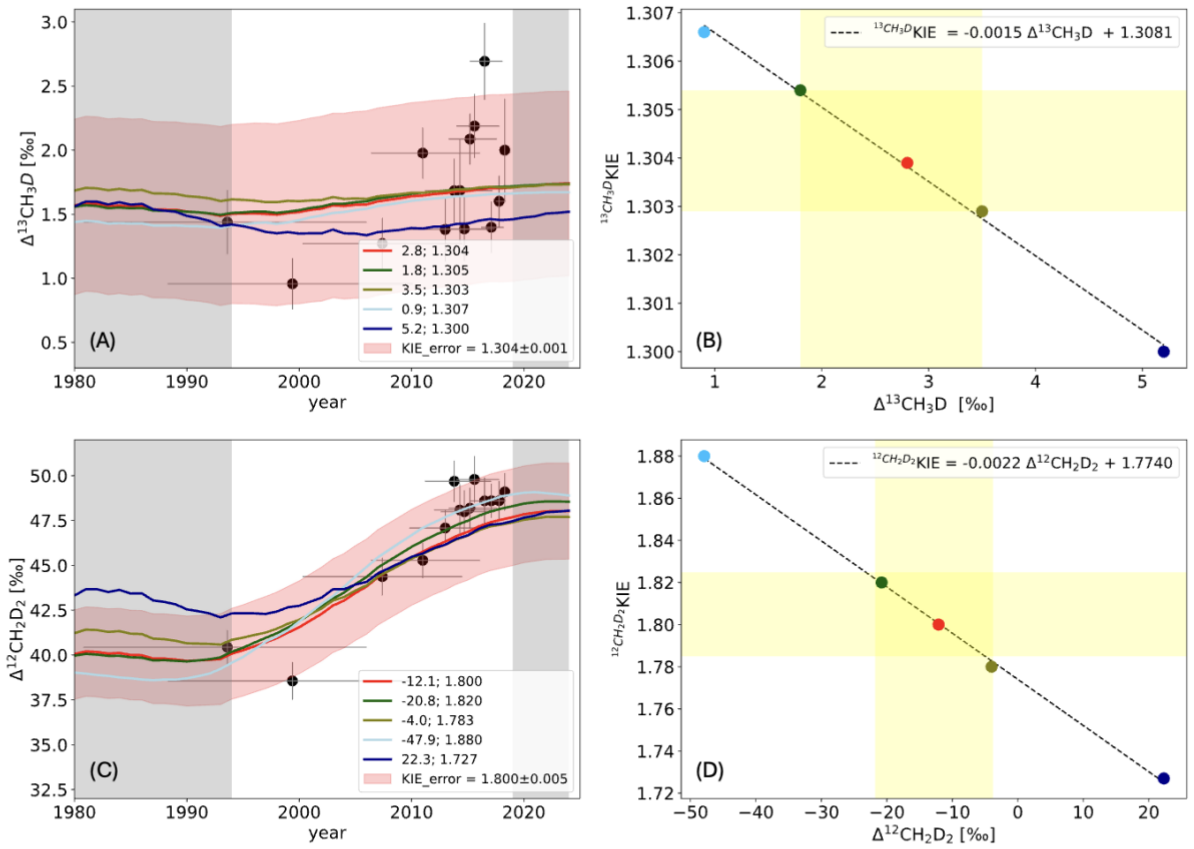

Fig. S9. The mutual constraint between the sink reaction KIE and the total source $\Delta^{13}CH_3D$ and $\Delta^{12}CH_2D_2$ for the Northern Hemisphere. (A) and **(C)**: Model outputs for different combinations of total source $\Delta^{13}CH_3D$ and $\Delta^{12}CH_2D_2$ signatures and the corresponding sink KIEs that fit the observed trends from the firn air measurements. Each combination is distinguished by a colour, as given in the legend (source mixture composition, sink KIE). The black circles represent the firn air samples measured in this study. **(B)** and **(D):** The linear relationship between the simulated total source $\Delta^{13}CH_3D$ and $\Delta^{12}CH_2D_2$ signatures (x-axis) and corresponding sink KIEs (y-axis). The vertically shaded yellow region highlights the most probable range of values for $\Delta^{13}CH_3D$ and $\Delta^{12}CH_2D_2$ deduced from existing datasets, and the horizontal, yellow-shaded region shows the required range of KIE values to fit the observation data.

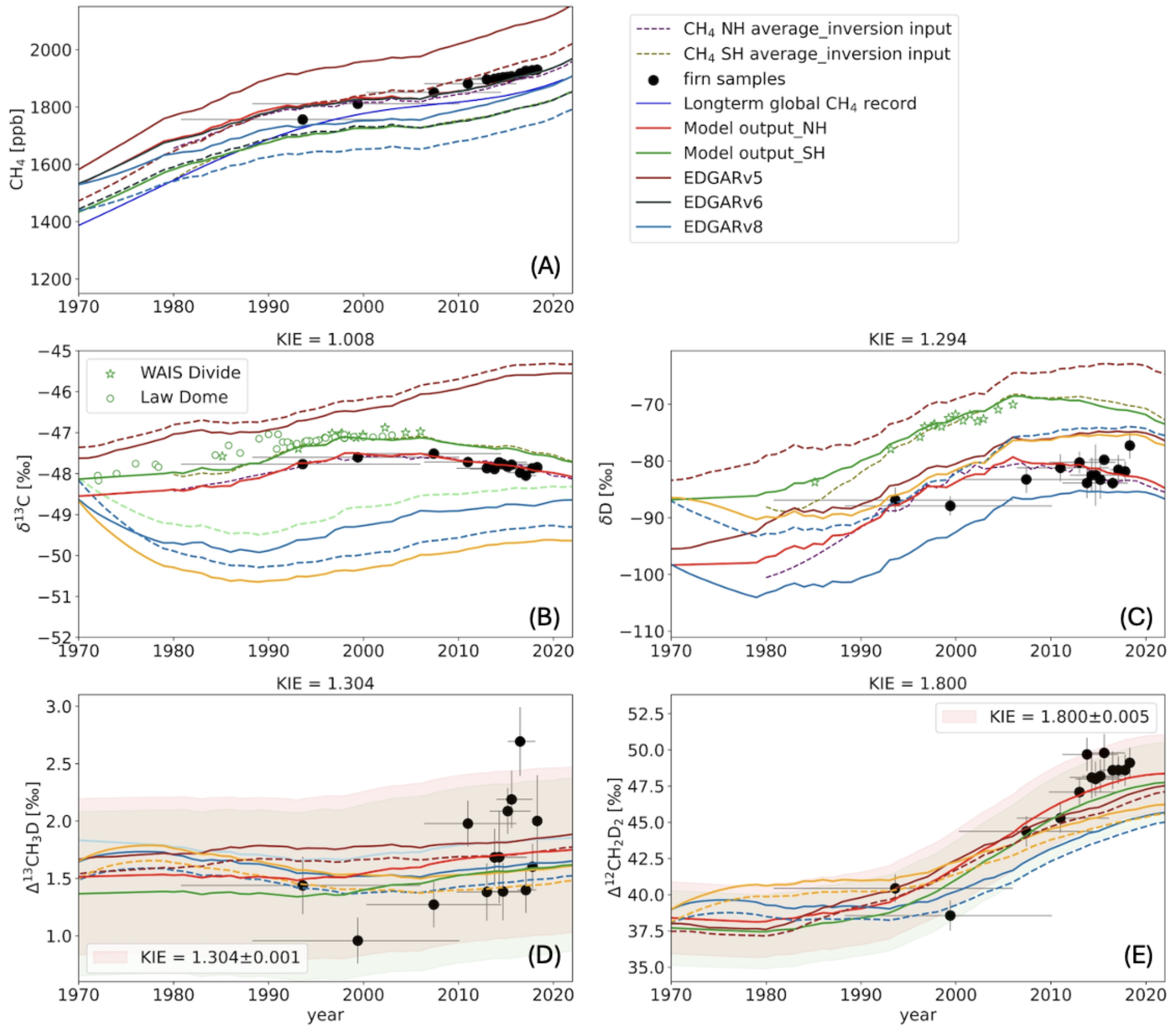

Fig. S10. Measured and modelled temporal evolution of atmospheric CH_4 mole fractions and isotopic signatures, with different EDGAR emission scenarios. (A) CH_4 mole fraction, **(B)** δ^{13}C, **(C)** δD, **(D)** $\Delta^{13}CH_3D$, and **(E)** $\Delta^{12}CH_2D_2$. Measurements from this study are shown in black circles. Measurements from previous studies for the SH (*4,21*) are shown in green circles and stars. Model output of the two-box forward model (this study) from 1970 to 2024 using different EDGAR emission scenarios (v5.0-brown, v6.0-darkblue, v8.0-lightblue), is shown in solid lines for NH and dashed lines for SH. The sink reaction KIEs for $^{13}CH_4$, $^{12}CH_3D$, $^{13}CH_3D$, and $^{12}CH_2D_2$ are given as legends in (B)-(E). The red and green shaded areas in (D) and (E) show the sensitivity of the KIEs used (±0.001 for $\Delta^{13}CH_3D$ and ±0.005 for $\Delta^{12}CH_2D_2$) for NH and SH, respectively.

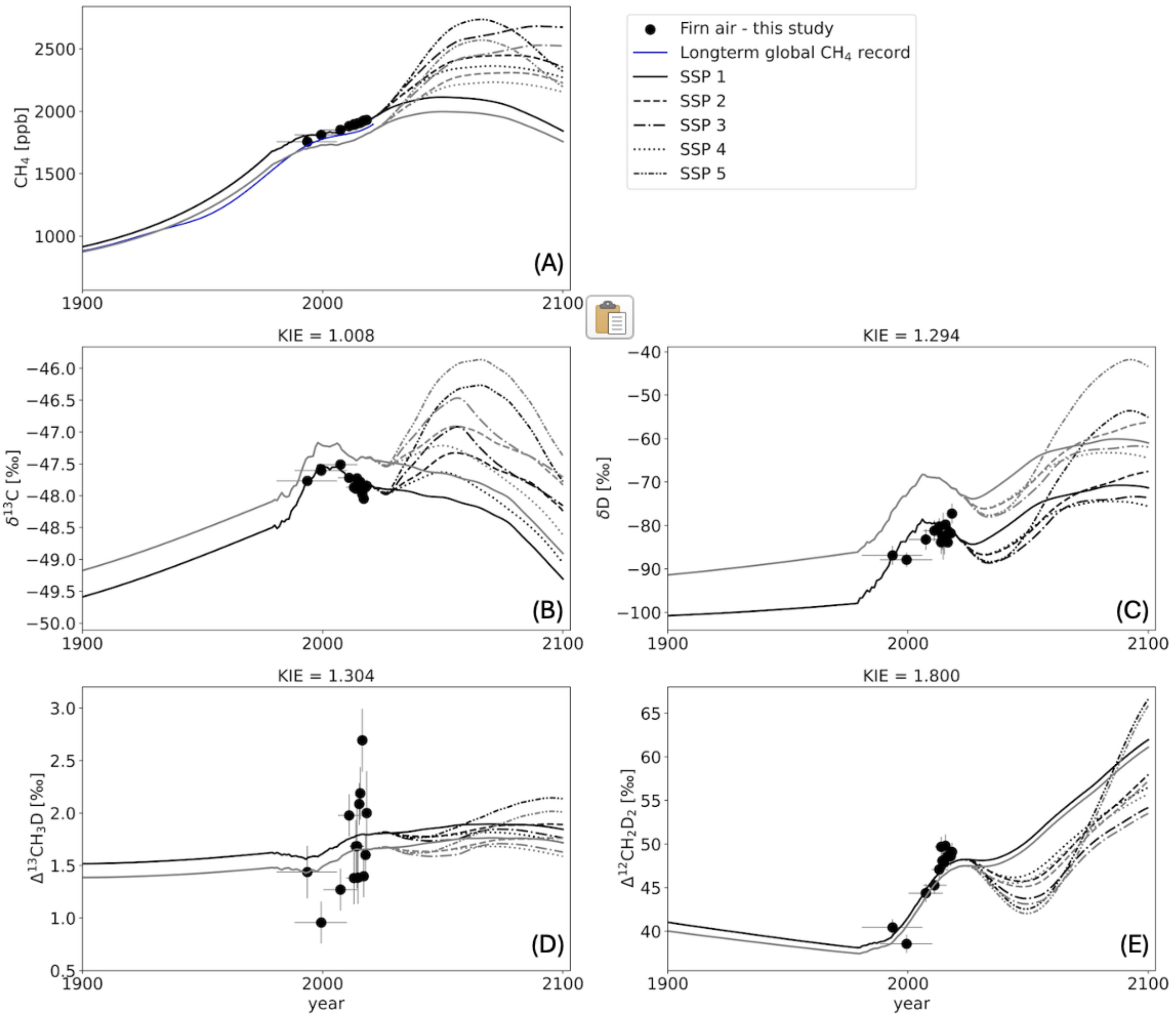

Fig. S11. Measured and modelled temporal evolution of atmospheric CH_4 mole fractions and isotopic signatures, with different IPCC SSP emission scenarios. (A) CH_4 mole fraction, **(B)** $\delta^{13}C$, **(C)** δD, **(D)** $\Delta^{13}CH_3D$, and **(E)** $\Delta^{12}CH_2D_2$. Measurements from this study are shown in black circles. Model output of the two-box forward model (this study) from 1900 to 2100 using different SSP emission scenarios (SSP 1 – solid line, SSP 2 – dashed line, SSP 3 – dash-dotted line, SSP 4 – dotted line, SSP 5 – dash-dot-dotted line), are shown in black lines for NH and grey lines for SH. The sink reaction KIEs for $^{13}CH_4$, $^{12}CH_3D$, $^{13}CH_3D$, and $^{12}CH_2D_2$ are given as legends in (B)-(E).

Tables and captions

Table S1: **Summary of measurement results of the firn samples analysed in this study.** Sampling depth, measured CH_4 mole fraction, $\delta^{13}C$, δD, $\Delta^{13}CH_3D$, and $\Delta^{12}CH_2D_2$ of the firn-

trapped air samples presented in this study. All the $\delta^{13}C$ measurements have an uncertainty of around 0.1 ‰.

Sample ID	Depth (m)	Volume of gas extracted (L)	Amount of CH_4 (μmol)	CH_4 mole fraction (ppb)	$\delta^{13}C$ (‰)	δD (‰)	$\Delta^{13}CH_3D$ (‰)	$\Delta^{12}CH_2D_2$ (‰)
1	0	350	27.3	1910.0	-47.69	-79.2 (±2.3)	2.0 (±0.4)	49.1 (±1.1)
3	11.78	465	36.7	1931.4	-47.65	-83.7 (±1.8)	1.6 (±0.2)	48.6 (±1.1)
6	23.25	535	42.2	1930.8	-47.83	-83.4 (±2.6)	1.4 (±0.2)	48.6 (±1.0)
18	33.01	280	22.3	1947.0	-47.79	-85.8 (±1.1)	2.7 (±0.3)	48.6 (±1.3)
16	43.44	350	27.4	1912.2	-47.61	-81.7 (±0.5)	2.2 (±0.3)	49.8 (±1.3)
13	48.2	560	43.7	1905.8	-47.66	-85.2 (±3.4)	2.1 (±0.2)	48.2 (±1.2)
12	51.8	315	24.6	1907.2	-47.60	-85.4 (±5.4)	1.4 (±0.3)	48.0 (±1.2)
11	54.07	250	19.6	1912.5	-47.56	-84.4 (±3.1)	1.7 (±0.4)	48.1 (±1.5)
14	55.75	425	33.1	1904.7	-47.73	-85.8 (±2.7)	1.7 (±0.3)	49.7 (±1.2)
15	57.65	425	33.0	1898.9	-47.73	-82.2 (±1.9)	1.4 (±0.3)	47.1 (±1.0)
10	59.18	555	42.7	1882.1	-47.63	-83.2 (±2.4)	2.0 (±0.2)	45.3 (±1.0)
9	60.86	475	36.0	1851.2	-47.50	-85.3 (±2.4)	1.3 (±0.2)	44.4 (±1.1)
8	62.92	525	39.0	1818.4	-47.83	-90.2 (±1.7)	1.0 (±0.2)	38.6 (±1.1)

7	64.22	500	36.2	1771.4	-48.32	-89.5 (±2.2)	1.5 (±0.3)	40.5 (±1.0)

Table S2: **Measurement results after corrections for molecular diffusion and gravitational settling in firn layers.** Mean age, age at 15% and 85% of the age distribution, and the corrected isotopic signatures of each firn air sample using the CH_4 IGE-GIPSA firn air model that accounted for diffusion and gravitation. All the $\delta^{13}C$ measurements have an uncertainty of around 0.1 ‰.

Depth (m)	Mean age	Age at 15%	Age at 85%	Corrected $\delta^{13}C$ (‰)	Corrected δD (‰)	Corrected $\Delta^{13}CH_3D$ (‰)	Corrected $\Delta^{12}CH_2D_2$ (‰)
0	2018.3	n.a.	n.a.	-47.69	-79.2 (±2.3)	2.0 (±0.4)	49.1 (±1.1)
11.78	2017.8	2018.4	2017.5	-47.71	-83.7 (±1.8)	1.6 (±0.2)	48.6 (±1.1)
23.25	2017.1	2018.2	2016.2	-47.88	-83.4 (±2.6)	1.4 (±0.2)	48.6 (±1.0)
33.01	2016.5	2018.1	2015.2	-47.81	-85.8 (±1.1)	2.7 (±0.3)	48.6 (±1.3)
43.44	2015.6	2017.8	2014.0	-47.63	-81.7 (±0.5)	2.2 (±0.3)	49.8 (±1.3)
48.2	2015.2	2017.6	2013.3	-47.68	-85.2 (±3.4)	2.1 (±0.2)	48.2 (±1.2)
51.8	2014.7	2017.4	2012.6	-47.62	-85.4 (±5.4)	1.4 (±0.3)	48.0 (±1.2)
54.07	2014.3	2017.2	2012.0	-47.57	-84.4 (±3.1)	1.7 (±0.4)	48.1 (±1.5)
55.75	2013.8	2017.1	2011.2	-47.74	-85.8 (±2.7)	1.7 (±0.3)	49.7 (±1.2)
57.65	2013.0	2016.8	2009.8	-47.72	-82.2 (±1.9)	1.4 (±0.3)	47.1 (±1.0)
59.18	2011.0	2016.1	2006.4	-47.57	-83.1 (±2.4)	2.0 (±0.2)	45.3 (±1.0)
60.86	2007.4	2014.5	2000.3	-47.36	-85.2 (±2.4)	1.3 (±0.2)	44.4 (±1.1)
62.92	1999.4	2010.1	1988.3	-47.45	-89.8 (±1.7)	1.0 (±0.2)	38.6 (±1.1)
64.22	1993.6	2006	1980.8	-47.61	-88.8 (±2.2)	1.4 (±0.3)	40.4 (±1.0)

Table S3: **Isotopic signature input for the two-box forward model.** Isotopic signatures of $\delta^{13}C$, δD, and $\Delta^{13}CH_3D$ and $\Delta^{12}CH_2D_2$ of the source categories used as the input for the forward model. $\delta^{13}C$ and δD are specified separately for the NH and SH.

Sources/Sinks	$\delta^{13}C$ SH (‰)	$\delta^{13}C$ NH (‰)	δD SH (‰)	δD NH (‰)	$\Delta^{13}CH_3D$ (‰)	$\Delta^{12}CH_2D_2$ (‰)
Wetlands	-58.3	-63.9	-297.0	-339.0	1.7	-36.0
Agriculture	-64.0	-63.0	-302.0	-309.0	-0.2	-39.8
Waste	-54.5	-54.5	-292.2	-292.2	1.2	-15.0
Fossil	-44.0	-44.0	-192.0	-194.0	3.6	7.6
Pyrogenic	-22.3	-22.4	-213.0	-183.0	2.5	-9.5

REFERENCES

1. S. Szopa, V. Naik, B. Adhikary, P. Artaxo, T. Berntsen, W. D. Collins, S. Fuzzi, L. Gallardo, A. Kiendler-Scharr, Z. Klimont, H. Liao, N. Unger, P. Zanis, "Short-lived climate forcers," in *Climate Change 2021: The Physical Science Basis. Contribution of Working Group I to the Sixth Assessment Report of the Intergovernmental Panel on Climate Change,* V. Masson-Delmotte, P. Zhai, A. Pirani, S. L. Connors, C. Péan, S. Berger, N. Caud, Y. Chen, L. Goldfarb, M. I. Gomis, M. Huang, K. Leitzell, E. Lonnoy, J. B. R. Matthews, T. K. Maycock, T. Waterfield, O. Yelekçi, R. Yu, B. Zhou, Eds. (Cambridge Univ. Press, 2021), pp. 817–922.

2. P. M. Forster, C. Smith, T. Walsh, W. F. Lamb, R. Lamboll, C. Cassou, M. Hauser, Z. Hausfather, J. Y. Lee, M. D. Palmer, K. von Schuckmann, A. B. A. Slangen, S. Szopa, B. Trewin, J. Yun, N. P. Gillett, S. Jenkins, H. D. Matthews, K. Raghavan, A. Ribes, J. Rogelj, D. Rosen, X. Zhang, M. Allen, L. Aleluia Reis, R. M. Andrew, R. A. Betts, A. Borger, J. A. Broersma, S. N. Burgess, L. Cheng, P. Friedlingstein, C. M. Domingues, M. Gambarini, T. Gasser, J. Gütschow, M. Ishii, C. Kadow, J. Kennedy, R. E. Killick, P. B. Krummel, A. Liné, D. P. Monselesan, C. Morice, J. Mühle, V. Naik, G. P. Peters, A. Pirani, J. Pongratz, J. C. Minx, M. Rigby, R. Rohde, A. Savita, S. I. Seneviratne, P. Thorne, C. Wells, L. M. Western, G. R. van der Werf, S. E. Wijffels, V. Masson-Delmotte, P. Zhai, Indicators of Global Climate Change 2024: Annual update of key indicators of the state of the climate system and human influence. *Earth Syst. Sci. Data* **17**, 2641–2680 (2025).

3. X. Lan, K. W. Thoning, E. J. Dlugokencky, Trends in globally-averaged CH_4, N_2O, and SF_6 determined from NOAA Global Monitoring Laboratory measurements, version 2026-06, NOAA Global Monitoring Laboratory (2022); https://doi.org/10.15138/P8XG-AA10.

4. E. G. Nisbet, M. R. Manning, E. J. Dlugokencky, S. E. Michel, X. Lan, T. Röckmann, H. A. C. Denier van der Gon, J. Schmitt, P. I. Palmer, M. N. Dyonisius, Y. Oh, R. E. Fisher, D. Lowry, J. L. France, J. W. C. White, G. Brailsford, T. Bromley, Atmospheric methane: Comparison between methane's record in 2006–2022 and during glacial terminations. *Global Biogeochem. Cycles* **37**, e2023GB007875 (2023).

5. A. L. Rice, C. L. Butenhoff, D. G. Teama, F. H. Röger, M. A. K. Khalil, R. A. Rasmussen, Atmospheric methane isotopic record favors fossil sources flat in 1980s and 1990s with recent increase. *Proc. Natl. Acad. Sci. U.S.A.* **113**, 10791–10796 (2016).

6. M. Rigby, S. A. Montzka, R. G. Prinn, J. W. C. White, D. Young, S. O'Doherty, M. F. Lunt, A. L. Ganesan, A. J. Manning, P. G. Simmonds, P. K. Salameh, C. M. Harth, J. Mühle, R. F. Weiss, P. J. Fraser, L. P. Steele, P. B. Krummel, A. McCulloch, S. Park, Role of atmospheric oxidation in recent methane growth. *Proc. Natl. Acad. Sci. U.S.A.* **114**, 5373–5377 (2017).

7. A. J. Turner, C. Frankenberg, P. O. Wennberg, D. J. Jacob, Ambiguity in the causes for decadal trends in atmospheric methane and hydroxyl. *Proc. Natl. Acad. Sci. U.S.A.* **114**, 5367–5372 (2017).

8. B. T. Uveges, R. W. Howarth, J. P. Sparks, Fossil fuel methane emissions likely underestimated in a model based on atmospheric $\delta^{13}C$ trends. *Proc. Natl. Acad. Sci. U.S.A.* **122**, e2507837122 (2025).

9. P. M. J. Douglas, D. A. Stolper, J. M. Eiler, A. L. Sessions, M. Lawson, Y. Shuai, A. Bishop, O. G. Podlaha, A. A. Ferreira, E. V. Santos Neto, M. Niemann, A. S. Steen, L. Huang, L. Chimiak, D. L. Valentine, J. Fiebig, A. J. Luhmann, W. E. Seyfried, G. Etiope, M. Schoell, W. P. Inskeep, J. J. Moran, N. Kitchen, Methane clumped isotopes: Progress and potential for a new isotopic tracer. *Org. Geochem.* **113**, 262–282 (2017).

10. E. D. Young, I. E. Kohl, B. S. Lollar, G. Etiope, D. Rumble III, S. Li, M. A. Haghnegahdar, E. A. Schauble, K. A. McCain, D. I. Foustoukos, C. Sutcliffe, O. Warr, C. J. Ballentine, T. C. Onstott, H. Hosgormez, A. Neubeck, J. M. Marques, I. Pérez-Rodríguez, A. R. Rowe, D. E. LaRowe, C. Magnabosco, L. Y. Yeung, J. L. Ash, L. T. Bryndzia, The relative abundances of resolved $^{12}CH_2D_2$ and $^{13}CH_3D$ and mechanisms controlling isotopic bond ordering in abiotic and biotic methane gases. *Geochim. Cosmochim. Acta* **203**, 235–264 (2017).

11. M. A. Haghnegahdar, J. Sun, N. Hultquist, N. D. Hamovit, N. Kitchen, J. Eiler, S. Ono, S. A. Yarwood, A. J. Kaufman, R. R. Dickerson, A. Bouyon, C. Magen, J. Farquhar, Tracing sources of atmospheric methane using clumped isotopes. *Proc. Natl. Acad. Sci. U.S.A.* **120**, e2305574120 (2023).

12. M. A. Haghnegahdar, N. Hultquist, N. D. Hamovit, S. A. Yarwood, A. Bouyon, A. J. Kaufman, J. Sun, C. Magen, J. Farquhar, A better understanding of atmospheric methane sources using $^{13}CH_3D$ and $^{12}CH_2D_2$ clumped isotopes. *J. Geophys. Res. Biogeosci.* **129**, e2024JG008172 (2024).

13. M. Sivan, T. Röckmann, C. van der Veen, M. E. Popa, Extraction, purification, and clumped isotope analysis of methane ($\Delta^{13}CDH_3$ and $\Delta^{12}CD_2H_2$) from sources and the atmosphere. *Atmos. Meas. Tech.* **17**, 2687–2705 (2024).

14. J. Sun, C. Magen, M. A. Haghnegahdar, J. Liu, J. M. Fernandez, J. Farquhar, Constraining wetland and landfill methane emission signatures through atmospheric methane clumped isotopologue measurements. *J. Geophys. Res. Biogeosci.* **130**, e2024JG008249 (2025).

15. J. Westhoff, J. Freitag, A. Orsi, P. Martinerie, I. Weikusat, M. Dyonisius, X. Faïn, K. Fourteau, T. Blunier, Combining traditional and novel techniques to increase our understanding of the lock-in depth of atmospheric gases in polar ice cores – Results from the EastGRIP region. *The Cryosphere* **18**, 4379–4397 (2024).

16. D. F. Ferretti, J. B. Miller, J. W. White, D. M. Etheridge, K. R. Lassey, D. C. Lowe, C. M. Macfarling Meure, M. F. Dreier, C. M. Trudinger, T. D. van Ommen, R. L. Langenfelds, Unexpected changes to the global methane budget over the past 2000 years. *Science* **309**, 1714–1717 (2005).

17. C. J. Sapart, P. Martinerie, E. Witrant, J. Chappellaz, R. S. W. van de Wal, P. Sperlich, C. van der Veen, S. Bernard, W. T. Sturges, T. Blunier, J. Schwander, D. Etheridge, T. Röckmann, Can the carbon isotopic composition of methane be reconstructed from multi-site firn air measurements? *Atmos. Chem. Phys.* **13**, 6993–7005 (2013).

18. M. A. Haghnegahdar, E. A. Schauble, E. D. Young, A model for $^{12}CH_2D_2$ and $^{13}CH_3D$ as complementary tracers for the budget of atmospheric CH_4. *Global Biogeochem. Cycles* **31**, 1387–1407 (2017).

19. E. Chung, T. Arnold, Potential of clumped isotopes in constraining the global atmospheric methane budget. *Global Biogeochem. Cycles* **35**, e2020GB006883 (2021).

20. B. Dasgupta, S. Pandey, S. Houweling, M. Menoud, C. van der Veen, J. Miller, B. Riddell-Young, S. Englund Michel, P. Sperlich, S. Morimoto, R. Fujita, I. Levin, C. Veidt, S. Platt, C. Groot Zwaaftink, C. Lund Myhre, C. Woolley Maisch, R. Fisher, E. G. Nisbet, J. France, R. Moss, N. Warwick, T. Röckmann, Global methane emission estimates from a dual-isotope inversion: New constraints from δD-CH_4. *EGUsphere* **2025**, 1–21 (2025).

21. J. A. Mischler, T. A. Sowers, R. B. Alley, M. Battle, J. R. McConnell, L. Mitchell, T. Popp, E. Sofen, M. K. Spencer, Carbon and hydrogen isotopic composition of methane over the last 1000 years. *Global Biogeochem. Cycles* **23**, 10.1029/2009GB003460 (2009).

22. P. P. Tans, A note on isotopic ratios and the global atmospheric methane budget. *Global Biogeochem. Cycles* **11**, 77–81 (1997).

23. B. F. Thornton, G. Etiope, S. Schwietzke, A. V. Milkov, R. W. Klusman, A. Judd, D. Z. Oehler, Conflicting estimates of natural geologic methane emissions. *Elementa Sci. Anthrop.* **9**, 00031 (2021).

24. G. Etiope, K. R. Lassey, R. W. Klusman, E. Boschi, Reappraisal of the fossil methane budget and related emission from geologic sources. *Geophys. Res. Lett.* **35**, L09307 (2008).

25. B. Hmiel, V. V. Petrenko, M. N. Dyonisius, C. Buizert, A. M. Smith, P. F. Place, C. Harth, R. Beaudette, Q. Hua, B. Yang, I. Vimont, S. E. Michel, J. P. Severinghaus, D. Etheridge, T. Bromley, J. Schmitt, X. Faïn, R. F. Weiss, E. Dlugokencky, Preindustrial $^{14}CH_4$ indicates greater anthropogenic fossil CH_4 emissions. *Nature* **578**, 409–412 (2020).

26. M. N. Dyonisius, V. V. Petrenko, A. M. Smith, Q. Hua, B. Yang, J. Schmitt, J. Beck, B. Seth, M. Bock, B. Hmiel, I. Vimont, J. A. Menking, S. A. Shackleton, D. Baggenstos, T. K. Bauska, R. H. Rhodes, P. Sperlich, R. Beaudette, C. Harth, M. Kalk, E. J. Brook, H. Fischer, J. P. Severinghaus, R. F. Weiss, Old carbon reservoirs were not important in the deglacial methane budget. *Science* **367**, 907–910 (2020).

27. A. R. Whitehill, L. M. T. Joelsson, J. A. Schmidt, D. T. Wang, M. S. Johnson, S. Ono, Clumped isotope effects during OH and Cl oxidation of methane. *Geochim. Cosmochim. Acta* **196**, 307–325 (2017).

28. T. Gierczak, R. K. Talukdar, S. C. Herndon, G. L. Vaghjiani, A. R. Ravishankara, Rate coefficients for the reactions of hydroxyl radicals with methane and deuterated methanes. *J. Phys. Chem. A* **101**, 3125–3134 (1997).

29. Global Methane Pledge (European Commission and United States, 2021). https://www.globalmethanepledge.org/.

30. Intergovernmental Panel on Climate Change, *Climate Change 2022 – Impacts, Adaptation and Vulnerability: Working Group II Contribution to the Sixth Assessment Report of the Intergovernmental Panel on Climate Change* (Cambridge Univ. Press, 2023).

31. M. Menoud, C. van der Veen, J. Necki, J. Bartyzel, B. Szénási, M. Stanisavljević, I. Pison, P. Bousquet, T. Röckmann, Methane (CH_4) sources in Krakow, Poland: Insights from isotope analysis. *Atmos. Chem. Phys.* **21**, 13167–13185 (2021).

32. J. Sun, M. A. Haghnegahdar, J. M. Fernandez, C. Magen, J. Farquhar, Controls on concentrations and clumped isotopologues of vehicle exhaust methane. *PLOS ONE* **20**, e0315304 (2025).

33. J. E. Mak, C. A. M. Brenninkmeijer, Compressed air sample technology for isotopic analysis of atmospheric carbon monoxide. *J. Atmos. Oceanic Tech.* **11**, 425–431 (1994).

34. M. Battle, M. Bender, T. Sowers, P. P. Tans, J. H. Butler, J. W. Elkins, J. T. Ellis, T. Conway, N. Zhang, P. Lang, A. D. Clarket, Atmospheric gas concentrations over the past century measured in air from firn at the South Pole. *Nature* **383**, 231–235 (1996).

35. C. Buizert, P. Martinerie, V. V. Petrenko, J. P. Severinghaus, C. M. Trudinger, E. Witrant, J. L. Rosen, A. J. Orsi, M. Rubino, D. M. Etheridge, L. P. Steele, C. Hogan, J. C. Laube, W. T. Sturges, V. A. Levchenko, A. M. Smith, I. Levin, T. J. Conway, E. J. Dlugokencky, P. M. Lang, K. Kawamura, T. M. Jenk, J. W. C. White, T. Sowers, J. Schwander, T. Blunier, Gas transport in firn: Multiple-tracer characterisation and model intercomparison for NEEM, Northern Greenland. *Atmos. Chem. Phys.* **12**, 4259–4277 (2012).

36. P. Martinerie, E. Nourtier-Mazauric, J. M. Barnola, W. T. Sturges, D. R. Worton, E. Atlas, L. K. Gohar, K. P. Shine, G. P. Brasseur, Long-lived halocarbon trends and budgets from atmospheric chemistry modelling constrained with measurements in polar firn. *Atmos. Chem. Phys.* **9**, 3911–3934 (2009).

37. C. M. Trudinger, D. M. Etheridge, P. J. Rayner, I. G. Enting, G. A. Sturrock, R. L. Langenfelds, Reconstructing atmospheric histories from measurements of air composition in firn. *J. Geophys. Res. Atmos.* **107**, ACH 15-1–ACH 15-13 (2002).

38. E. Witrant, P. Martinerie, C. Hogan, J. C. Laube, K. Kawamura, E. Capron, S. A. Montzka, E. J. Dlugokencky, D. Etheridge, T. Blunier, W. T. Sturges, A new multi-gas constrained model of trace gas non-homogeneous transport in firn: Evaluation and behaviour at eleven polar sites. *Atmos. Chem. Phys.* **12**, 11465–11483 (2012).

39. T. Umezawa, S. Sugawara, K. Kawamura, I. Oyabu, S. J. Andrews, T. Saito, S. Aoki, T. Nakazawa, Towards reconstructing the Arctic atmospheric methane history over the 20th century: Measurement and modelling results for the North Greenland Ice Core Project firn. *Atmos. Chem. Phys.* **22**, 6899–6917 (2022).

40. L. Y. Yeung, E. D. Young, E. A. Schauble, Measurements of $^{18}O^{18}O$ and $^{17}O^{18}O$ in the atmosphere and the role of isotope-exchange reactions. *J. Geophys. Res. Atmos.* **117**, D18306 (2012).

41. L. Y. Yeung, L. T. Murray, P. Martinerie, E. Witrant, H. Hu, A. Banerjee, A. Orsi, J. Chappellaz, Isotopic constraint on the twentieth-century increase in tropospheric ozone. *Nature* **570**, 224–227 (2019).

42. C. Buizert, T. Sowers, T. Blunier, Assessment of diffusive isotopic fractionation in polar firn, and application to ice core trace gas records. *Earth Planet. Sci. Lett.* **361**, 110–119 (2013).

43. J. R. Worden, A. A. Bloom, S. Pandey, Z. Jiang, H. M. Worden, T. W. Walker, S. Houweling, T. Röckmann, Reduced biomass burning emissions reconcile conflicting estimates of the post-2006 atmospheric methane budget. *Nat. Commun.* **8**, 2227 (2017).

44. C. J. Sapart, G. Monteil, M. Prokopiou, R. S. W. van de Wal, J. O. Kaplan, P. Sperlich, K. M. Krumhardt, C. van der Veen, S. Houweling, M. C. Krol, T. Blunier, T. Sowers, P. Martinerie, E. Witrant, D. Dahl-Jensen, T. Rockmann, Natural and anthropogenic variations in methane sources during the past two millennia. *Nature* **490**, 85–88 (2012).

45. S. Naus, S. A. Montzka, S. Pandey, S. Basu, E. J. Dlugokencky, M. Krol, Constraints and biases in a tropospheric two-box model of OH. *Atmos. Chem. Phys.* **19**, 407–424 (2019).

46. B. Dasgupta, M. Menoud, C. van der Veen, I. Levin, C. Veidt, H. Moossen, S. Englund Michel, P. Sperlich, S. Morimoto, R. Fujita, T. Umezawa, S. M. Platt, C. G. Zwaaftink, C. L. Myhre, R. Fisher, D. Lowry, E. Nisbet, J. France, C. Woolley Maisch, G. Brailsford, R. Moss, D. Goto, S. Pandey, S. Houweling, N. Warwick, T. Röckmann, Harmonisation of methane isotope ratio measurements from different laboratories using atmospheric samples. *EGUsphere* **2025**, 1–21 (2025).

47. R. Fujita, H. Graven, G. Zazzeri, B. Hmiel, V. V. Petrenko, A. M. Smith, S. E. Michel, S. Morimoto, Global fossil methane emissions constrained by multi-isotopic atmospheric methane histories. *J. Geophys. Res. Atmos.* **130**, e2024JD041266 (2025).

48. IPCC, *2019 Refinement to the 2006 IPCC Guidelines for National Greenhouse Gas Inventories* (IPCC, 2019).

49. IPCC, *IPCC Guidelines for National Greenhouse Gas Inventories*, H. S. Eggleston, L. Buendia, K. Miwa, T. Ngara, K. Tanabe, Eds. (IPCC TSU NGGIP, IGES, 2006).

50. N. Thiagarajan, H. Xie, C. Ponton, N. Kitchen, B. Peterson, M. Lawson, M. Formolo, Y. Xiao, J. Eiler, Isotopic evidence for quasi-equilibrium chemistry in thermally mature natural gases. *Proc. Natl. Acad. Sci. U.S.A.* **117**, 3989 (2020).

51. L. Taenzer, J. Labidi, A. L. Masterson, X. Feng, D. Rumble, E. D. Young, W. D. Leavitt, Low $\Delta_{12}CH_2D_2$ values in microbialgenic methane result from combinatorial isotope effects. *Geochim. Cosmochim. Acta* **285**, 225–236 (2020).

52. J. Labidi, E. D. Young, T. Giunta, I. E. Kohl, J. Seewald, H. Tang, M. D. Lilley, G. L. Früh-Green, Methane thermometry in deep-sea hydrothermal systems: Evidence for re-ordering of doubly-substituted isotopologues during fluid cooling. *Geochim. Cosmochim. Acta* **288**, 248–261 (2020).

53. Y. Shuai, P. M. J. Douglas, S. Zhang, D. A. Stolper, G. S. Ellis, M. Lawson, M. D. Lewan, M. Formolo, J. Mi, K. He, G. Hu, J. M. Eiler, Equilibrium and non-equilibrium controls on the abundances of clumped isotopologues of methane during thermogenic formation in laboratory experiments: Implications for the chemistry of pyrolysis and the origins of natural gases. *Geochim. Cosmochim. Acta* **223**, 159–174 (2018).

54. Y. Shuai, G. Etiope, S. Zhang, P. M. J. Douglas, L. Huang, J. M. Eiler, Methane clumped isotopes in the Songliao Basin (China): New insights into abiotic vs. biotic hydrocarbon formation. *Earth Planet. Sci. Lett.* **482**, 213–221 (2018).

55. D. A. Stolper, A. M. Martini, M. Clog, P. M. Douglas, S. S. Shusta, D. L. Valentine, A. L. Sessions, J. M. Eiler, Distinguishing and understanding thermogenic and biogenic sources of methane using multiply substituted isotopologues. *Geochim. Cosmochim. Acta* **161**, 219–247 (2015).

56. E. D. Young, D. Rumble, P. Freedman, M. Mills, A large-radius high-mass-resolution multiple-collector isotope ratio mass spectrometer for analysis of rare isotopologues of O_2, N_2, CH_4 and other gases. *Int. J. Mass Spectrom.* **401**, 1–10 (2016).

57. D. L. Eldridge, R. Korol, M. K. Lloyd, A. C. Turner, M. A. Webb, T. F. Miller, D. A. Stolper, Comparison of experimental vs theoretical abundances of $^{13}CH_3D$ and $^{12}CH_2D_2$ for isotopically equilibrated systems from 1 to 500 °C. *ACS Earth and Space Chem.* **3**, 2747–2764 (2019).

58. D. T. Wang, D. S. Gruen, B. S. Lollar, K. U. Hinrichs, L. C. Stewart, J. F. Holden, A. N. Hristov, J. W. Pohlman, P. L. Morrill, M. Könneke, K. B. Delwiche, E. P. Reeves, C. N. Sutcliffe, D. J. Ritter, J. S. Seewald, J. C. McIntosh, H. F. Hemond, M. D. Kubo, D. Cardace, T. M. Hoehler, S. Ono, Methane cycling. Nonequilibrium clumped isotope signals in microbial methane. *Science* **348**, 428–431 (2015).

59. D. T. Wang, P. V. Welander, S. Ono, Fractionation of the methane isotopologues $^{13}CH_4$, $^{12}CH_3D$, and $^{13}CH_3D$ during aerobic oxidation of methane by Methylococcus capsulatus (Bath). *Geochim. Cosmochim. Acta* **192**, 186–202 (2016).

60. S. Ono, D. T. Wang, D. S. Gruen, B. Sherwood Lollar, M. S. Zahniser, B. J. McManus, D. D. Nelson, Measurement of a doubly substituted methane isotopologue, $^{13}CH_3D$, by tunable infrared laser direct absorption spectroscopy. *Anal. Chem.* **86**, 6487–6494 (2014).

61. D. A. Stolper, M. Lawson, C. L. Davis, A. A. Ferreira, E. V. S. Neto, G. S. Ellis, M. D. Lewan, A. M. Martini, Y. Tang, M. Schöll, A. L. Sessions, J. M. Eiler, Formation temperatures of thermogenic and biogenic methane. *Science* **344**, 1500–1503 (2014).

62. D. A. Stolper, A. L. Sessions, A. A. Ferreira, E. V. S. Neto, A. Schimmelmann, S. S. Shusta, D. L. Valentine, J. M. Eiler, Combined ^{13}C–D and D–D clumping in methane: Methods and preliminary results. *Geochim. Cosmochim. Acta* **126**, 169–191 (2014).

63. D. S. Gruen, D. T. Wang, M. Könneke, B. D. Topçuoğlu, L. C. Stewart, T. Goldhammer, J. F. Holden, K.-U. Hinrichs, S. Ono, Experimental investigation on the controls of clumped isotopologue and hydrogen isotope ratios in microbial methane. *Geochim. Cosmochim. Acta* **237**, 339–356 (2018).

64. D. T. Wang, E. P. Reeves, J. M. McDermott, J. S. Seewald, S. Ono, Clumped isotopologue constraints on the origin of methane at seafloor hot springs. *Geochim. Cosmochim. Acta* **223**, 141–158 (2018).

65. Y. Gonzalez, D. D. Nelson, J. H. Shorter, J. B. McManus, C. Dyroff, M. Formolo, D. T. Wang, C. M. Western, S. Ono, Precise measurements of $^{12}CH_2D_2$ by tunable infrared laser direct absorption spectroscopy. *Anal. Chem.* **91**, 14967–14974 (2019).

66. P. M. J. Douglas, R. Gonzalez Moguel, K. M. Walter Anthony, M. Wik, P. M. Crill, K. S. Dawson, D. A. Smith, E. Yanay, M. K. Lloyd, D. A. Stolper, J. M. Eiler, A. L. Sessions, Clumped isotopes link older carbon substrates with slower rates of methanogenesis in northern lakes. *Geophys. Res. Lett.* **47**, (2020).

67. T. Giunta, E. D. Young, O. Warr, I. Kohl, J. L. Ash, A. Martini, S. O. C. Mundle, D. Rumble, I. Pérez-Rodríguez, M. Wasley, D. E. LaRowe, A. Gilbert, B. Sherwood Lollar, Methane sources and sinks in continental sedimentary systems: New insights from paired clumped isotopologues 13CH3D and 12CH2D2. *Geochim. Cosmochim. Acta* **245**, 327–351 (2019).

68. T. Giunta, E. D. Young, J. Labidi, P. Sansjofre, D. Jézéquel, J.-P. Donval, C. Brandily, L. Ruffine, Extreme methane clumped isotopologue bio-signatures of aerobic and anaerobic methanotrophy: Insights from the Lake Pavin and the Black Sea sediments. *Geochim. Cosmochim. Acta* **338**, 34–53 (2022).

69. G. Dong, H. Xie, M. Formolo, M. Lawson, A. Sessions, J. Eiler, Clumped isotope effects of thermogenic methane formation: Insights from pyrolysis of hydrocarbons. *Geochim. Cosmochim. Acta* **303**, 159–183 (2021).

70. S. Ono, J. H. Rhim, D. S. Gruen, H. Taubner, M. Kölling, G. Wegener, Clumped isotopologue fractionation by microbial cultures performing the anaerobic oxidation of methane. *Geochim. Cosmochim. Acta* **293**, 70–85 (2021).

71. N. Zhang, G. T. Snyder, M. Lin, M. Nakagawa, A. Gilbert, N. Yoshida, R. Matsumoto, Y. Sekine, Doubly substituted isotopologues of methane hydrate ($^{13}CH_3D$ and $^{12}CH_2D_2$): Implications for methane clumped isotope effects, source apportionments and global hydrate reservoirs. *Geochim. Cosmochim. Acta* **315**, 127–151 (2021).

72. S. J. E. Krause, J. Liu, E. D. Young, T. Treude, $\Delta_{13}CH_3D$ and $\Delta_{12}CH_2D_2$ signatures of methane aerobically oxidized by Methylosinus trichosporium with implications for deciphering the provenance of methane gases. *Earth Planet. Sci. Lett.* **593**, 117681 (2022).

73. J. Liu, R. L. Harris, J. L. Ash, J. G. Ferry, S. J. E. Krause, J. Labidi, D. Prakash, B. Sherwood Lollar, T. Treude, O. Warr, E. D. Young, Reversibility controls on extreme methane clumped isotope signatures from anaerobic oxidation of methane. *Geochim. Cosmochim. Acta* **348**, 165–186 (2023).

74. J. Liu, T. Treude, O. R. Abbasov, E. E. Baloglanov, A. A. Aliyev, C. M. Harris, W. D. Leavitt, E. D. Young, Clumped isotope evidence for microbial alteration of thermogenic methane in terrestrial mud volcanoes. *Geology* **52**, 22–26 (2023).

75. J. H. Rhim, S. Ono, Combined carbon, hydrogen, and clumped isotope fractionations reveal differential reversibility of hydrogenotrophic methanogenesis in laboratory cultures. *Geochim. Cosmochim. Acta* **335**, 383–399 (2022).

76. E. Lalk, A. Velez, S. Ono, Methane clumped isotopologue variability from ebullition in a mid-latitude lake. *ACS Earth Space Chem.* **8**, 689–701 (2024).

77. J. Sun, M. A. Haghnegahdar, C. Magen, J. Farquhar, *2022 Goldschmidt Conference* (GOLDSCHMIDT, Hawaii, 2022).

78. J. Sun, J. Farquhar, M. A. Haghnegahdar, *Goldschmidt 2023 Conference* (Lyon, 2023).

79. M. A. Haghnegahdar, A. Bouyon, N. Hamovit, C. Magen, C. J. Celarie, P. Megonigal, S. Yarwood, G. L. Noyce, J. Farquhar, *AGU Fall 2023* (Chicago, 2023).

80. S. M. Defratyka, J. M. Fernandez, G. A. Adnew, G. Dong, P. M. J. Douglas, D. L. Eldridge, G. Etiope, T. Giunta, M. A. Haghnegahdar, A. N. Hristov, N. Hultquist, I. Vadillo, J. Jautzy, J. H. Kim, J. Labidi, E. Lalk, W. Leavitt, J. Li, L. H. Lin, J. Liu, L. Ojeda, S. Ono, J. H. Rhim, T. Röckmann, B. Sherwood Lollar, M. Sivan, J. Sun, G. T. Ventura, D. T. Wang, E. D. Young, N. Zhang, T. Arnold, Global inventory of doubly substituted isotopologues of methane ($\Delta_{13}CH_3D$ and $\Delta_{12}CH_2D_2$). *Earth Syst. Sci. Data* **17**, 6889–6910 (2025).

81. K. Riahi, R. Schaeffer, J. Arango, K. Calvin, C. Guivarch, T. Hasegawa, K. Jiang, E. Kriegler, R. Matthews, G. P. Peters, A. Rao, S. Robertson, A. M. Sebbit, J. Steinberger, M. Tavoni, D. P. v. Vuuren, "Mitigation pathways compatible with long-term goals," in *Climate Change 2022: Mitigation of Climate Change. Contribution of Working Group III to the Sixth Assessment Report of the Intergovernmental Panel on Climate Change*, Intergovernmental Panel on Climate Change, Ed. (Cambridge Univ. Press, 2023), pp. 295–408.

82. E. Byers, V. Krey, E. Kriegler, K. Riahi, R. Schaeffer, J. Kikstra, R. Lamboll, Z. Nicholls, M. Sanstad, C. Smith, K.-I. v. d. Wijst, A. A. Khourdajie, F. Lecocq, J. Portugal-Pereira, Y. Saheb, A. Strømann, H. Winkler, C. Auer, E. Brutschin, M. Gidden, P. Hackstock, M. Harmsen, D. Huppmann, P. Kolp, C. Lepault, J. Lewis, G. Marangoni, E. Müller-Casseres, R. Skeie, M. Werning, K. Calvin, P. Forster, C. Guivarch, T. Hasegawa, M. Meinshausen, G. Peters, J. Rogelj, B. Samset, J. Steinberger, M. Tavoni, D. v. Vuuren, AR6 Scenarios Database hosted by IIASA, version 1.0, International Institute for Applied Systems Analysis (2022); https://doi.org/10.5281/zenodo.5886911.

83. E. Stehfest, D. van Vuuren, T. Kram, L. Bouwman, R. Alkemade, M. Bakkenes, H. Biemans, A. Bouwman, M. den Elzen, J. Janse, P. Lucas, J. van Minnen, C. Müller, A. Prins, "Integrated assessment of global environmental change with IMAGE 3.0. Model description and policy applications" (PBL Netherlands Environmental Assessment Agency, 2014).
